# Availability of residents and preceptors for interprofessional practices: mixed methods study *

**DOI:** 10.1590/1518-8345.7374.4395

**Published:** 2024-10-25

**Authors:** André Lucas Maffissoni, Jussara Gue Martini, Daniele Delacanal Lazzari, Carine Vendruscolo, Marina da Silva Sanes, Paula Bresolin

**Affiliations:** ^1^ Universidade Federal de Santa Catarina, Florianópolis, SC, Brazil; ^2^ Scholarship holder at the Conselho Nacional de Desenvolvimento Científico e Tecnológico (CNPq), Brazil.; ^3^ Universidade do Estado de Santa Catarina, Departamento de Enfermagem, Chapecó, SC, Brazil.

**Keywords:** Internship and Residency, Interprofessional Education, Interdisciplinary Placement, Interprofessional Relations, Interdisciplinary Communication, Health Education

## Abstract

to evaluate the level of availability of residents and preceptors for interprofessional practices.

mixed methods study of the concurrent triangulation type, carried out with residents and preceptors of a hospital Multiprofessional Residency in Health.

more than 90% (n=186) of participants are highly available for interprofessional practices. However, practices instituted during undergraduate studies and the professional profile of those involved, especially with regard to the lack of communication between professions and openness to dialogue, were indicated as difficulties in implementing interprofessionality in the scenario investigated, which may negatively influence the availability of residents and preceptors.

although residents and preceptors have high availability for the development of interprofessionality, certain factors make its implementation difficult, hindering collaborative work.

## Highlights

**(1)** The level of availability influences hospital interprofessional practices.

**(2)** Difficulties in interprofessional communication impede collaborative practices.

**(3)** There is resistance to the adoption of interprofessional practices related to education.

**(4)** Residencies are important spaces to develop interprofessional practices.

## Introduction

 Interprofessional health practices encompass a set of actions carried out by multiprofessional teams, aiming at the collective elaboration and execution of qualified care ^(^ 1 ^)^ . Given the complexity inherent to hospital scenarios, both nationally and internationally, several strategies are proposed to encourage this collaborative process, such as Interprofessional Education (IPE) and Collaborative Practice (CP). 

 IPE materializes through the co-learning of students from different areas, aiming to increase collaboration and, consequently, optimize health outcomes. The CP, on the other hand, seeks the convergence of health professionals with varied expertise, involving patients, families, caregivers and communities, with the purpose of generating care aimed at patient safety ^(^ 1 ^-^ 2 ^)^ . Although they have conceptual differences, these strategies prove to be interdependent, since collaboration at work is driven by mutual learning, and, conversely, collaborative learning develops within a unified perspective of action. 

 Collaborative practices can be fostered through curricular subjects or even through proposals to be operationalized in different emphases, such as specific meetings with common themes, tutorials or other deliberate activities that, based on critical thinking, establish strategies to be discussed and actions coordinated together for each patient ^(^ 3 ^)^ . However, this collaboration does not occur without obstacles, especially in the hospital context, since, despite advances in the provision of care in these cases, there remains a tendency towards uniprofessional care, centered on the biomedical model, often anchored in pathology ^(^ 4 ^-^ 6 ^)^ . 

 The hospital Multiprofessional Residency in Health (MRH) began in 2010, with the aim of contributing to the education of professionals for the *Sistema Único de Saúde* (SUS), outlining new possibilities for learning and work, in a space historically marked by fragmentation of care. The merits of MRH in improving hospital care are undeniable, although difficulties persist in implementing strategies related to CP ^(^ 7 ^-^ 10 ^)^ . 

 Therefore, it is essential to investigate whether MRH residents, preceptors and tutors are willing to develop teaching, learning and care, based on an interprofessional perspective ^(^ 11 ^)^ . It is desirable that these professionals develop collaborative activities, aware of the importance of sharing responsibilities regarding care demands. National and international studies have been dedicated to measuring availability as a way to identify problems and promote the development of interprofessional practices ^(^ 12 ^-^ 14 ^)^ . 

 The importance of interprofessional education in clinical learning environments is grounded in the opportunity to develop and integrate skills and competencies in the authentic sociocultural context of the healthcare environments in which residents will work ^(^ 5 ^)^ . Thus, measuring availability, even through self-reported instruments, can provide evidence that supports personalized interventions to promote collaborative skills in teamwork, in order to achieve common goals ^(^ 7 ^)^ . The way residents perceive actual practice may differ from stereotypes or beliefs they held before the collaborative experience. Additionally, the practice location may not match their perception of what an interprofessional practice would be like ^(^ 7 ^)^ . 

 Factors related to teaching can affect the ability to train collaborative professionals, such as socialization, the learning context and the development of teaching staff. In this way, what is taught in this context must converge in terms of content, that is, collaborative skills must stimulate the recognition of the responsibilities of each of the professionals ^(^ 8 ^)^ . In a systematic review, which aimed to evaluate the effectiveness of interprofessional education for Nursing and Medical professionals and students regarding their attitudes, skills, knowledge and behaviors, it indicated that the evidence on this effectiveness is inconclusive, demonstrating the need for other studies on the subject ^(^ 9 ^)^ . Besides, a systematic review that aimed to investigate the effects of interprofessional education on collaborative practice among health professionals demonstrated that it is a viable approach to improving attitudes and mutual respect among these professionals, and indicated that further research is needed, considering the development and incorporation of IPE into curricula, the low cost and benefits of this learning method ^(^ 6 ^)^ . 

This investigation was based on the following research question: what is the degree of availability of residents and preceptors for interprofessional practices, and what factors influence it? The objective of the study was to evaluate the level of availability of residents and preceptors for interprofessional practices.

## Method

 Mixed methods study of the concurrent triangulation type, in which quantitative and qualitative data are collected concomitantly and then mixed. This mixed study design gives the same weight to the two steps (QUAN + QUAL), as both have the same relevance for achieving the objectives ^(^ 15 ^)^ . Data mixing was carried out by integration, a process in which elements are merged and generate information that supports mutually to understand the object of the study ^(^ 15 ^)^ . The choice of this approach was influenced by prior knowledge of the residency program, as it is developed in the hospital where the researchers work, therefore recognizing the complex interactions between residents, preceptors, professors and tutors. In this way, upon being aware of the empirical intention of having interprofessional practices, continually manifested in classes and tutorials by everyone involved in the teaching and learning processes, it was decided to attribute the same weight to the quantitative and qualitative steps, aiming to investigate the phenomenon concomitantly. 

 The quantitative step was carried out based on an observational study, of a descriptive-analytical cross-sectional type, with data collection based on the application of two steps, designed in accordance with the Strengthening the Reporting of Observational Studies in Epidemiology (STROBE). In the qualitative step, exploratory-descriptive research was developed, with interviews, guided by the Consolidated Criteria for Reporting Qualitative Research (COREQ). To comply with the methodological rigor of the mixed study, the Mixed Methods Appraisal Tool (MMAT) ^(^ 16 ^)^ was used. 

### Scenario

 Study carried out in the *Residência Integrada Multiprofissional em Saúde* (RIMS) program at the *Universidade Federal de Santa Catarina* (UFSC), developed at the *Hospital Universitário Polydoro Ernani de São Thiago* (HU-UFSC), city of Florianópolis, Santa Catarina, Brazil. 

RIMS was created at HU-UFSC in 2010, based on a partnership between the Departments of Nursing, Pharmacy, Physiotherapy, Speech Therapy, Nutrition, Dentistry, Psychology and Social Services, with the HU board of directors. Residents are admitted annually, with a selection process offering 36 vacancies in three areas of concentration: Urgency and Emergency Care, High Complexity Healthcare and Women’s and Children’s Healthcare.

### Population

Study participants were first- and second-year residents from all professions involved in RIMS, and preceptors who provided direct supervision to residents. It is noteworthy that the researchers involved in this study do not work as preceptors in the residency. Some of the researchers act as tutors or teachers, and these groups did not compose the sample. It should also be noted that the latter did not participate in data collection at any time.

### Selection criteria

The following criteria were adopted for participating in the study: 1) residents: be regularly enrolled in the program; and 2) preceptors: have any connection (public servant or employee) with the hospital institution and carry out direct supervision activities with residents. Those residents and preceptors who were away were excluded.

### Definition of the sample and participants

The definition of the sample in the quantitative step was carried out using different strategies for residents and preceptors. In the case of residents, the number of people enrolled in 2022 was 36 first-year residents (R1) and 27 second-year residents (R2), totaling 63 residents. For preceptors, the average number of professionals on a work schedule was calculated over a period of three months (February, March and April 2022). This was due to high absenteeism and professional turnover. The initial sample calculation indicated 204 eligible professionals and, after the inclusion criteria, it was composed of 165 professionals.

The qualitative step of the study consisted of 26 participants (among those who participated in the first step). The interviews were carried out in a private location within the hospital institution, by the main researcher. The average interview time was 27 minutes, with length varying between 18 and 38 minutes. The transcribed interviews were sent individually to each participant, to validate the written content, making changes when requested by the interviewee.

### Instruments used

 To collect quantitative data, the Readiness for Interprofessional Learning Scale (RIPLS) ^(^ 11 ^)^ and the Jefferson Scale of Attitudes Toward Interprofessional Collaboration (JeffSATIC) ^(^ 17 ^)^ were used, both validated for Brazilian Portuguese. 

 The RIPLS, which assesses the availability of subjects for the development of IPE, is composed of 27 items and divided into three factors: Factor 1 is called Teamwork and Collaboration and is related to positive attitudes towards availability for shared learning, confidence and respect between professional areas; Factor 2 is titled Professional Identity and relates to specific aspects of professions and professional autonomy; and Factor 3 is called Patient-Centered Healthcare, relating to positive attitudes aimed at health needs from the patient’s perspective. The overall RIPLS score ranges between 27 and 135 points, with 70, 40 and 25 being the maximum score for Factors 1, 2 and 3, respectively ^(^ 11 ^)^ . 

 The JeffSATIC scale, adapted and validated in Brazil in 2016, is aimed at analyzing attitudes related to CP, and is composed of 20 questions, with 140 being the maximum score ^(^ 17 ^)^ . In both scales, availability for IPE and CP is directly related to the score, so the more points the respondent obtains, the greater their availability for IPE. It is noteworthy that RIPLS and JeffSATIC were complemented with a section dedicated to characterizing the participants. 

To collect qualitative data, the interviews were guided by a semi-structured script, with questions about how participants experienced interprofessional practices in their daily lives, openness to development, difficulties, potential and aspects subject to change. This script was not validated, but a pilot test was carried out with former preceptors and former residents, who did not compose the sample and whose data were not included in the study.

### Data collection

Quantitative and qualitative data were collected between May 2022 and February 2023. Quantitative data were collected by a hired scholarship holder, a master’s student at the same institution, unrelated to MRH and who was trained on the instruments and how to apply them. Participants were free to fill out the printed forms upon receipt or take them home and return them later. After defining the sample, the distribution and application of the instruments began, simultaneously with the interviews, which were carried out by the main researcher.

In the qualitative step, to participate in the interviews, residents and preceptors were contacted by email and/or telephone. The invitations were made randomly, but the representativeness of the number of preceptors and residents in relation to professional categories was observed. To guarantee impartiality, a randomization strategy was used based on specific criteria, without direct interference from researchers in the selection of interviewees. This was done manually, without the use of randomization software, to ensure a representative and varied sample. There were no refusals to participate in the interviews.

### Data processing and analysis

 For the analysis of quantitative data, the IBM SPSS Statistics 25.0 software was used. Descriptive statistics were used based on measures of central tendency (mean or median) and dispersion (standard deviation or interquartile range) for the analysis of continuous data, while the analysis of absolute and relative frequencies was used for categorical data. For the bivariate analysis, data normality was checked using the Kolmogorov-Smirnov test with Lilliefors adjustment on all categories (sociodemographic data and RIPLS and JeffSATIC scales), indicating the adoption of the alternative hypothesis of non-normality (p < 0.05) for all. For comparisons between participants, the non-parametric Mann-Whitney U tests were used for two independent variables and the Kruskal-Wallis test for three or more independent variables, and, in the latter, when H0 was rejected, Dunn’s *post hoc* analysis. 

Regarding the scales, it is noteworthy that items 10, 11, 12, 17, 19 and 21 of RIPLS and items 3, 5, 8, 9, 12, 15, 16 and 19 of JeffSATIC refer to negative attitudes related to IPE and CP, and, therefore, for data analysis, the scores were inverted. To accurately determine participants’ availability for IPE and CP, it was decided to fragment the score into quartiles. In this way, each 25% of the global score possible to achieve on both scales corresponded to one of the following levels of availability for learning and interprofessional practice, which are: not available, low availability, moderate availability and high availability.

Regarding age, for analysis purposes, preceptors were divided into a group up to 39 years old and another over 39 years old, considering that the median age was 39 years old. For residents, a group between 20 and 29 years old and another between 30 and 39 years old were considered, as there were no residents under 20 years old and over 39 years old in the sample.

To organize the qualitative data, the interviews were audio-recorded and transcribed in full. For the reliability of the qualitative data, three of the researchers in this study discussed and validated the themes and subthemes arising from the participants’ statements, collected from semi-structured interviews. The option to use a mixed methodology also contributed to the reliability of the qualitative data, as it was possible to identify the mix of quantitative results obtained concurrently, continually expanding the understanding of the phenomenon investigated.

 The information from the interviews was analyzed qualitatively through thematic content analysis, proposed by Bardin ^(^ 18 ^)^ . The analytical process was carried out in three phases: pre-analysis, material exploration and data processing. The ATLAS.ti software was used for the three steps of content analysis, generating 29 codes (recording units) and 350 quotations (context units). From these units, the intersection of data interpretations and thematic categorization were performed. 

### Ethical aspects

 The study was approved by the Ethics Committee of the *Universidade Federal de Santa Catarina* under opinion n.º 5,256,168. All principles presented by Resolutions n.º 466/2012 and n.º 510/2016 of the *Conselho Nacional de Saúde* were complied with. All steps of the research were previously explained to the participants and their optional participation in any of them was clarified. All participants were aware of and signed the Free and Informed Consent Form. To preserve their identity, participants were identified with the letter R for resident or P for preceptor and the respective acronym of the professional category to which they belonged. When there was more than one participant from the same profession, they were numbered with Arabic numerals (1 or 2). 

## Results

 A total of 146 preceptors and 58 residents participated in the research. A 95% Confidence Index (CI) and a 5% margin of error were respected. Regarding sociodemographic data, there was a predominance of female participants, both in the group of preceptors and in the group of residents. The minimum age of the participants was 22 years, and the maximum was 62 years. The eight professions that make up the MRH had similar representation in terms of the number of participants, with the exception of Nursing, which had the largest number when compared to all the others, since it is the profession with the largest number of vacancies in the program. In Table 1 it is possible to observe the details of the participants’ characteristics. 

**Table 1:** - Sociodemographic characteristics of residents and preceptors according to frequency for categorical variables and median for continuous variables. Florianópolis, SC, Brazil, 2023

**Variable**	N* (%) or median (minimum-maximum)	
	**Preceptors** (N*=146)	**Residents** (N*=58)
**Age**	39 [27.0 – 62.0]	25 [22.0 – 35.0]
**Gender**		
Male	19 (13.0%)	12 (20.7%)
Female	127 (87.0%)	46 (79.3%)
**Profession**		
Nursing	79 (54.1%)	16 (27.6%)
Speech Therapy	9 (6.2%)	03 (5.2%)
Psychology	10 (6.8%)	11 (19.0%)
Pharmacy	11 (7.5%)	7 (12.1%)
Social Work	13 (8.9%)	11 (19.0%)
Physiotherapy	8 (5.5%)	2 (3.4%)
Nutrition	12 (8.2%)	5 (8.6%)
Dentistry	4 (2.7%)	3 (5.2%)
**MRH** ^†^ **concentration area**		
High Complexity	93 (63.7%)	37 (63.8%)
Urgency and Emergency	13 (8.9%)	11 (19.0%)
Women’s and Children’s Health	40 (27.4 %)	10 (17.2%)
**Time since graduation**		
Up to 2 years	NA ^‡^	26 (44.8%)
2 to 5 years	NA ^‡^	30 (51.7%)
More than 5 years	NA ^‡^	2 (3.4%)
**MRH** ^†^ **year**		
First year (R1)	NA ^‡^	35 (60.3%)
Second year (R2)	NA ^‡^	23 (39.7%)
**Professional experience**		
Up to 2 years	1 (0.7%)	NA ^‡^
3 and 5 years	8 (5.5%)	NA ^‡^
6 and 10 years	34 (23.3%)	NA ^‡^
More than 10 years	103 (70.5%)	NA ^‡^
**Time working as a preceptor**		
Up to 2 years	27 (18.5%)	NA ^‡^
Between 3 and 5 years	53 (36.3%)	NA ^‡^
Between 6 and 10 years	43 (29.5%)	NA ^‡^
More than 10 years	22 (15.1%)	NA ^‡^
No answer	1 (0.6%)	NA ^‡^

* N = Sample

^†^ RMS = Multiprofessional Residency in Health

^‡^ NA = Not applicable

 Regarding availability, all participants fell into the moderate and high availability percentiles, both for IPE, assessed by RIPLS, and for CP, assessed by JeffSATIC. For preceptors, the proportion in RIPLS was 90.41% (132) with high availability and 9.58% (14) with moderate availability; in JeffSATIC the proportion was 99.31% (145) with high availability and 0.68% (1) with moderate availability. For residents, the proportion in RIPLS was 94.82% (55) with high availability and 5.17% (3) with moderate availability; in JeffSATIC all 58 residents achieved high availability. Table 2 presents the overall scores of the two scales for residents and preceptors. 

**Table 2:** - Scores of Factors 1, 2 and 3 of the RIPLS* scale and overall score of the RIPLS* and JeffSATIC ^†^ scales. Florianópolis, SC, Brazil, 2023

	RIPLS* dimensions			RIPLS*	**JeffSATIC** ^†^
	**Fac 1** ^‡^	**Fac 2** ^§^	**Fac 3** ^||^	**Score**	**Score**
**Preceptors (N** ^¶^ **=146)**					
Mean	59.4	31.0	22.2	112.7	126.6
Standard Deviation	5.5	2.9	2.2	8.2	8.4
Median	60.0	31.0	23.0	113.0	128.0
Minimum score	43	22	16	89	100
Maximum score	69	39	25	130	140
**Residents (N** ¶ **=58)**					
Mean	61.0	31.1	22.7	114.9	128.7
Standard Deviation	4.0	2.9	2.2	6.9	7.6
Median	61.0	31.0	23.0	115.5	131.0
Minimum score	51	23	17	95	108
Maximum score	66	37	25	128	140

* RIPLS = *Readiness for Interprofessional Learning Scale*

^†^ JeffSATIC = Jefferson Scale of Attitudes Toward Interprofessional Collaboration

^‡^ Fat 1 **=** Factor 1

^§^ Fat 2 = Factor 2

^||^ Fat 3 = Factor 3

^¶^ N = Sample

When comparing preceptors from different professions, there were no suggestive differences in the RIPLS and JeffSATIC scores; however, a lower median was observed in the overall score of both scales in nurse preceptors (RIPLS = 112 and JeffSATIC = 122). The same occurred when tests were carried out comparing residents from different professions, however, the lowest medians observed in this case were in Social Services for RIPLS (109) and in Speech Therapy for JeffSATIC (123).

 Specifically for the time since graduation, there was a suggestive difference (P < 0.014) for Factor 1 of RIPLS - Teamwork and Collaboration. In the *post hoc* test, the difference was found between residents with up to 2 years since graduation and among those with more than 5 years. 

 When comparing the overall scores between preceptors and residents of the same profession, the results indicate differences in two professions, as can be seen in Table 3 . 

**Table 3:** - Comparison of the overall score on the RIPLS* and JeffSATIC ^†^ scales according to preceptors and residents of the same profession. Florianópolis, SC, Brazil, 2023

	RIPLS* dimensions			**Score**	
**Profession**	**Fac 1** ^‡^	**Fac 2** ^§^	**Fac 3** ^||^	RIPLS*	**JeffSATIC** ^†^
**NUR** ^¶^					
Preceptors	61.0 [45.0 – 67.0]	32.0 [24.0 – 39.0]	22.0 [16.0 – 25.0]	113.0 [96.0 – 130.0]	129.0 [100.0 – 140.0]
Residents	60.0 [54.0 – 66.0]	32.0 [23.0 – 36.0]	22.5 [17.0 – 25.0]	118.0 [95.0 – 125.0]	131.5 [112.0 – 140.0]
*P* value**	0.575	0.320	0.884	0.417	0.322
**PHA** ^††^					
Preceptors	60.0 [54.0 – 69.0]	31.0 [27.0 – 34.0]	22.0 [18.0 – 25.0]	111.0 [105.0 – 122.0]	126.0 [111.0 – 137.0]
Residents	60.0 [51.0 – 66.0]	30.0 [26.0 – 34.0]	23.0 [18.0 – 25.0]	112.0 [101.0 – 125.0]	122.0 [108.0 – 134.0]
*P* value**	0.930	0.596	0.479	0.930	0.536
**PHY** ^‡‡^					
Preceptors	61.0 [56.0 – 66.0]	31.0 [28.0 – 37.0]	23.5 [20.0 – 25.0]	117.5 [107.0 – 123.0]	130.0 [110.0 – 140.0]
Residents	64.5 [63.0 – 66.0]	32.0 [30.0 – 34.0]	22.5 [20.0 – 25.0]	119.0 [113.0 – 125.0]	129.0 [124.0 – 134.0]
*P* value**	0.400	0.711	1.000	0.533	1.000
**SPE** ^§§^					
Preceptors	58.0 [51.0 – 66.0]	31.0 [27.0 – 33.0]	23.0 [20.0 – 25.0]	112.0 [106.0 – 122.0]	124.0 [120.0 – 137.0]
Residents	64.0 [64.0 – 65.0]	35.0 [29.0 – 35.0]	24.0 [23.0 – 24.0]	122.0 [117.0 – 124.0]	134.0 [133.0 – 139.0]
*P* value**	0.064	0.373	0.373	**0.018**	**0.036**
**NUT** ^||||^					
Preceptors	56.0 [43.0 – 66.0]	29.5 [27.0 – 25.0]	22.5 [19.0 – 25.0]	110.0 [89.0 – 119.0]	123.0 [111.0 – 135.0]
Residents	64.0 [59.0 – 66.0]	31.0 [29.0 – 35.0]	25.0 [21.0 – 25.0]	120.0 [116.0 – 123.0]	133.0 [122.0 -140.0]
*P* value**	**0.004**	0.442	0.082	**0.001**	**0.037**
**DEN** ^¶¶^					
Preceptors	60.5 [58.0 – 66.0]	31.0 [29.0 – 33.0]	21.5 [20.0 – 23.0]	112.5 [109.0 – 121.0]	124.0 [118.0 – 131.0]
Residents	60.0 [55.0 – 61.0]	32.0 [30.0 – 34.0]	25.0 [20.0 – 25.0]	114.0 [110.0 – 118.0]	139.0 [133.0 – 140.0]
*P* value**	0.629	0.629	0.400	0.857	0.057
PSY***					
Preceptors	63.0 [49.0 – 66.0]	32.5 [29.0 – 35.0]	24.0 [21.0 – 25.0]	119.0 [103.0 – 123.0]	127.0 [116.0 – 138.0]
Residents	61.0 [57.0 – 66.0]	31.0 [27.0 – 37.0]	23.0 [20.0 – 25.0]	113.0 [109.0 – 128.0]	126.0 [117.0 – 135.0]
*P* value**	0.173	0.314	0.468	0.223	0.756
**SOC** ^†††^					
Preceptors	60.00 [49.0 – 66.0]	28.0 [22.0 – 35.0]	23.0 [20.0 – 25.0]	109.0 [98.0 – 125.0]	125.0 [109.0 – 140.0]
Residents	60.0 [55.0 – 66.0]	28.0 [26.0 – 33.0]	23.0 [19.0 – 25.0]	115.0 [100.0 – 124.0]	130.0 [111.0 – 139.0]
*P* value**	0.733	0.733	0.820	0.361	0.569

* RIPLS = *Readiness for Interprofessional Learning Scale*

^†^ JeffSATIC = Jefferson Scale of Attitudes Toward Interprofessional Collaboration

^‡^ Fat 1 **=** Fator 1

^§^ Fat 2 = Fator 2

^||^ Fat 3 = Fator 3

^¶^ NUR = Nursing

** Mann Whitney U test

^††^ PHA = Pharmacy

^‡‡^ PHY = Physiotherapy

^§§^ SPE = Speech Therapy

^||||^ NUT = Nutrition

^¶¶^ DEN = Dentistry

*** PSY = Psychology

^†††^ SOC = Social Service

The null hypothesis adopted was that the distribution of scores would be the same among residents and preceptors. This hypothesis was rejected for Speech Therapy in the RIPLS global score (U= 1.500; p < 0.018) and in the JeffSATIC global score (U= 2.500; p < 0.036). It was also rejected for Nutrition in the RIPLS Factor 1 score (U= 4.500; p < 0.004), RIPLS global score (U= 2.500; p < 0.001) and JeffSATIC global score (U= 10.500; p < 0.037).

The thematic analysis indicated that the undergraduate education process and the resistance of some professionals are two of the main opportunities to improve availability. The willingness for horizontal dialogue and collaboration of the multiprofessional team, both among preceptors and residents, were highlighted as the main potentialities.

The education of professionals, mostly from a uniprofessional perspective, plays a limiting role in interprofessional practices, due to the lack of recognition of the roles of colleagues from another professional category, as can be seen in the statements.

*We are still moving towards this* [interprofessional practices] *, it is not something specific to here, but it also results from our education, which is only focused on our professional category, we do not have knowledge of the technical aspects of other professionals so that they can take the actions we see them taking.* (P.SPE) 

*Teachers themselves have difficulty introducing our profession into classes in an interprofessional context. I understand this, because although we are all health professions, we are trained separately and there are different segments within each profession, so I understand the difficulty.* (R.DEN1) 

*One difficulty I have, especially in class, is that I do not have clinical education. We do not see anything about the body* [anatomy, physiology, etc.] *, and our education is isolated from that of all other health professionals. So sometimes I do not even understand what they are talking about, most of the things I have to ask about.* (R.SOC1) 

*We have difficulty establishing relationships with other professionals because it is very restricted to our area. In Dentistry, unfortunately, we have an education that does not involve other professionals, so we have the consequence of this difficulty in knowing how to work with everyone to meet the needs of patients.* (R.DEN2) 

*I see that we end up trying to explain all the time what we came here for. I see more that Psychology and Social Work really need to prove why they are there, people have difficulty in demanding Social Work or Psychology, largely due to the undergraduate education as well, which is more isolated from other professions.* (R.PSY1) 

Regarding the professional profile, influences occur due to the resistance of some professionals in developing collaboration for both teaching and work. There is an apprehension about sharing information and a tendency to act autonomously, especially when it comes to students or residents.

*There is a lot of resistance from people, it is very... I do not know if that is the right word, but selfishness. There are some professions that we notice that they do not want to share their knowledge because they are afraid that others know more than they do, this discourages us, how are we going to learn together like this?* (R.PHA1) 

*There was a nursing colleague who used to say, “I do not like students, I do not want anyone from the residency with me.” These professionals did things without the students, to do things faster, which ended up leaving them with no desire to learn.* (P.NUR1) 

*We see who is on duty, we look for someone we already know, someone we know has a friendlier, more sensitive, more collaborative attitude. Sometimes I prefer not to discuss the case depending on who the other professional is, because I know it will not evolve, they will not listen to me.* (P.SOC) 

On the other hand, easy access and horizontal dialogue influence the desire to learn how to work collaboratively in the availability. Effective dialogue and parity in the weight of contributions in the multiprofessional team support the will to act collaboratively.

*That is why it is important to have good access to the team. In the NICU, I can talk to the doctor and the nurses all the time. The team has a meeting system to discuss cases and procedures, and not just to discuss procedures, but to discuss changes.* (P.SOC) 

*Here I felt that we were able to communicate with the team and set goals together. The multiprofessional team of rehabilitation, physiotherapists, speech therapists, nutritionists, psychologists and nurses, which motivates us to want to share ideas.* (P.PHY1)**

*So, doctors, nurses and other professionals come to me to ask questions and exchange ideas. It is very open and relaxed, it is a good dialogue.* (R.PHY) 

*In the NICU, each case is discussed in depth with all professionals, it is all developed together, no decision is made without first discussing it with the speech therapist, nurses and social services. Everything is discussed in depth. We see how important each other is.* (R.NUR2) 

*There are people who are more willing, more accessible, easier to deal with, and with these people the work flows.* (P.NUR1) 

Undergraduate education influences the development of interprofessional practices, from the perspectives of residents and preceptors in Social Work and Psychology, who reported difficulties in working in the multiprofessional team, due to limitations in understanding the biological aspects of care and the lack of recognition of the importance of psychosocial aspects by other professions, such as Dentistry, whose education is focused on the anatomical and pathophysiological aspects of the mouth, and there is not much predisposition to dialogue with students from other professions in the health area.

**Figure 1:**
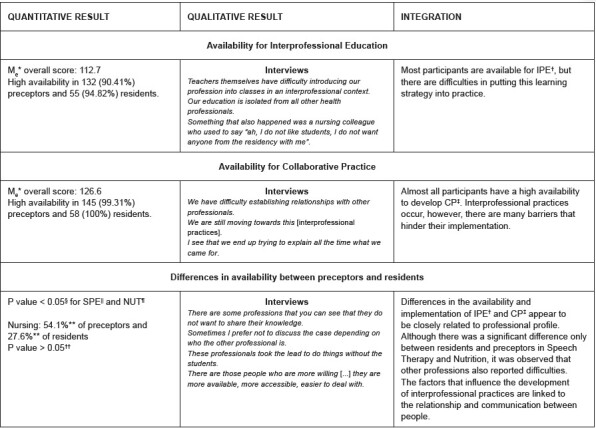
- Joint-display for integration of quantitative and qualitative data. Florianópolis, SC, Brazil, 2023

 For data integration and understanding, the joint-display technique was used, shown in Figure 1 . 

*M _e_ = Mean; ^†^ IPE = Interprofessional Education; ^‡^ CP = Collaborative Practice; §Mann-Whitney U test; ^||^ SPE = Speech Therapy; ¶NUT = Nutrition; **Highest frequency of responses; ^††^ Kruskal-Wallis test

## Discussion

 Interprofessional practices play an important role in fostering the sharing of experiences between multiprofessional teams and between residents from different professions, promoting patient-centered care and the convergence of areas of knowledge ^(^ 1 ^-^ 3 ^)^ . In the context of this study, the findings indicate a high availability of participants to develop interprofessional practices in educational and care frameworks. 

 It is worth noting that this high availability may be related to the context in which the participants are inserted, especially considering the environment of university hospitals, which are conducive to the implementation of innovations in the provision of care, due to their connections with universities, fostering teaching and research ^(^ 19 ^)^ . Additionally, MRH offers a differentiated approach to postgraduate education, allowing residents and preceptors the opportunity for simultaneous learning and practice, and encouraging collaboration between professionals from different areas ^(^ 8 ^-^ 9 ^,^ 19 ^)^ . Thus, awareness of the importance of collaboration in teaching and care can be intensified, promoting availability for interprofessional practices. 

 Despite this, participants indicate that willingness alone is not enough to implement consistent interprofessional practices in hospital MRH. This may be related to hospital routines, which still remain rigid, with professional roles delimited by historically defined boundaries, often marked by the absence of autonomy or responsibilities related to the technical execution of procedures. The predominance of the biomedical model still places diagnosis and cure as the focus, resulting in the overvaluation of medical practices and less autonomy and recognition of other professionals, which negatively impacts the appreciation of collaborative work ^(^ 19 ^)^ . 

 The biomedical model maintains its historical influence in the health field, not only in clinical care, but also in education. This study highlights that undergraduate education, except for a few isolated initiatives, often maintains a disciplinary and uniprofessional approach to knowledge, which can limit opportunities for students to learn from and with each other ^(^ 6 ^)^ . The lack of awareness of collaboration in undergraduate courses complicates the education of collaborative professionals. Furthermore, this gap can perpetuate a cycle of resistance or partial adoption of interprofessional practices, especially as these students have difficulty integrating interprofessional knowledge and actions in their future workplaces ^(^ 20 ^)^ . 

 However, attributing part of the difficulties of working interprofessionally to undergraduate education may mask the difficulties of understanding the limits established within each professional area, which seems to be more defined in Medicine, compared to other professions ^(^ 21 ^)^ . The idea of needing to learn another’s profession in order to then contribute interprofessionally conflicts with the need for multiple professionals in their own education to work together, each within their scope, in order to then collaborate with each other ^(^ 5 ^)^ . Another critical point for the development of IPE and CP, as indicated by the participants of this study, are certain professional characteristics, especially regarding communication, an essential tool in the health area ^(^ 2 ^,^ 22 ^)^ . Ineffective communication and a lack of openness to interprofessional dialogue compromise teaching and care, perpetuating the fragmentation of care activities and knowledge. 

 Considering a competency-based approach to interprofessional practice, communication emerges as a fundamental attribute ^(^ 23 ^-^ 24 ^)^ . Effective, empathetic and nonviolent communication not only facilitates interprofessional collaboration, but also creates healthy learning environments and safe care practices, minimizing errors and promoting care ^(^ 24 ^)^ . In this context, communication skills require training and improvement, enabling positive changes in the attitudes and behaviors of professionals and encouraging constant and welcoming dialogue within the MRH. 

This study contributes significantly to the advancement of knowledge in the health area, addressing a globally relevant topic. To implement interprofessional practices, it is essential, among other actions, to measure availability and understand the factors that influence the adherence of professionals and students to this approach. The MRH appears as a promising tool for incorporating these practices into the Brazilian hospital context, fully justifying its investigation.

Among the limitations of this study, we point out the absence of MRH teachers and tutors as participants, as well as patients and family members, whose inclusion could enrich the understanding of the phenomenon, especially in the qualitative context. Furthermore, the inclusion of medical professionals and residents would provide a comprehensive view of interprofessional dynamics and their impact on the availability to collaborate, as evaluating professionals in the multiprofessional residency without considering the role of the physician in approaching the patient indicates a selection bias, after all, the actions of the multiprofessional team include approaches from all professions in the health area. Additionally, residents and preceptors with more positive attitudes toward IPE may have been more likely to participate in the study, which may have influenced the results.

## Conclusion

The study demonstrated the availability for the development of interprofessional practices of residents and preceptors of a hospital Multiprofessional Residency in Health. The quantitative results indicated high availability for Interprofessional Education and Collaborative Practice among the participants and, based on the integration of the data collected concurrently, the qualitative results made it possible to understand the factors involved in this availability for IPE and CP.

Despite this, undergraduate education and the characteristics of the professionals involved, such as difficulties in communication between professions and openness to dialogue, are highlighted as difficulties in the development and maintenance of interprofessional practices. These difficulties can and should be observed from the perspective of opportunities, seeking to improve the communication skills of residents, preceptors and professionals in general, with a view to improving teaching and work processes within the scope of Multiprofessional Residencies in Health.
