# Supplementary material for: Availability of residents and preceptors for interprofessional practices: mixed methods study
Source: Rev Lat Am Enfermagem. 2024 Oct 25;32:e4394. doi: 10.1590/1518-8345.7374.4395 (PMC11526211; doi:10.1590/1518-8345.7374.4395)
Supplement: Translation

# Translation: Disponibilidad de residentes y preceptores para prácticas interprofesionales: estudio de métodos mixtos *

**DOI:** 10.1590/1518-8345.7374.4394

**Published:** 2024-10-03

**Authors:** André Lucas Maffissoni, Jussara Gue Martini, Daniele Delacanal Lazzari, Carine Vendruscolo, Marina da Silva Sanes, Paula Bresolin

**Affiliations:** ^1^ Universidade Federal de Santa Catarina, Florianópolis, SC, Brasil; ^2^ Bolsista do Conselho Nacional de Desenvolvimento Científico e Tecnológico (CNPq), Brasil.; ^3^ Universidade do Estado de Santa Catarina, Departamento de Enfermagem, Chapecó, SC, Brasil.

**Keywords:** Internado y Residencia, Educación Interprofesional, Prácticas Interdisciplinarias, Relaciones Interprofesionales, Comunicación Interdisciplinaria, Educación en Salud

## Abstract

evaluar el nivel de disponibilidad de residentes y de preceptores para prácticas interprofesionales.

estudio de métodos mixtos del tipo triangulación concomitante, realizado con residentes y preceptores de una Residencia Multiprofesional en Salud hospitalaria.

más del 90% (n=186) de los participantes tienen alta disponibilidad para prácticas interprofesionales. Sin embargo, las prácticas instituidas durante el pregrado y el perfil profesional de los involucrados, especialmente en lo que respecta a la falta de comunicación entre profesiones y la apertura al diálogo, fueron señalados como dificultades para implementar la interprofesionalidad en el escenario investigado, lo que podría influir negativamente en la disponibilidad de residentes y preceptores.

si bien residentes y preceptores tienen alta disponibilidad para el desarrollo de la interprofesionalidad, ciertos factores dificultan su implementación, obstaculizando el trabajo colaborativo.

## Destacados

**(1)** El nivel de disponibilidad influye en las prácticas interprofesionales hospitalarias.

**(2)** Dificultades en la comunicación interprofesional impiden prácticas colaborativas.

**(3)** Hay resistencias a la adopción de prácticas interprofesionales relacionadas con la formación.

**(4)** Residencias son espacios importantes para desarrollar prácticas interprofesionales.

## Introducción

 Las prácticas interprofesionales de salud engloban un conjunto de acciones realizadas por equipos multiprofesionales, encaminadas a la elaboración y ejecución colectiva de un cuidado calificado ^(^ 1 ^)^ . Dada la complejidad inherente a los escenarios hospitalarios, tanto a nivel nacional como internacional, se proponen varias estrategias para incentivar este proceso colaborativo, como la Educación Interprofesional (EIP) y la Práctica Colaborativa (PC). 

 La EIP se materializa por medio del coaprendizaje de estudiantes de diferentes áreas, con el objetivo de incrementar la colaboración y, en consecuencia, optimizar los resultados de salud. La PC, por su parte, tiene como objetivo reunir a profesionales de la salud con variada experiencia, involucrando a pacientes, familias, cuidadores y comunidades, con el propósito de generar cuidados orientados a la seguridad del paciente ^(^ 1 ^-^ 2 ^)^ . Aunque tienen diferencias conceptuales, estas estrategias demuestran ser interdependientes, ya que la colaboración en el trabajo está impulsada por el aprendizaje mutuo y, a la inversa, el aprendizaje colaborativo se desarrolla dentro de una perspectiva unificada de acción. 

 Las prácticas colaborativas pueden fomentarse por medio de materias curriculares o incluso por medio de propuestas a operacionalizar en diferentes énfasis, como reuniones específicas con temas comunes, tutorías u otras actividades deliberadas que, a partir del pensamiento crítico, establezcan estrategias a discutir y acciones en conjunto para cada paciente ^(^ 3 ^)^ . Sin embargo, esta colaboración no se produce sin obstáculos, especialmente en el contexto hospitalario, ya que, a pesar de los avances en la prestación de cuidados en estos entornos, persiste una tendencia hacia un cuidado uniprofesional, centrado en el modelo biomédico, a menudo anclado en la patología ^(^ 4 ^-^ 6 ^)^ . 

 La Residencia Multiprofesional en Salud (RMS) hospitalaria comenzó en 2010, con el objetivo de contribuir a la formación de profesionales para el *Sistema Único de Saúde* (SUS), perfilando nuevas posibilidades de aprendizaje y trabajo, en un espacio históricamente marcado por la fragmentación del cuidado. Los méritos de la RMS para mejorar el cuidado hospitalario son innegables, aunque persisten dificultades para implementar estrategias relacionadas con la PC ^(^ 7 ^-^ 10 ^)^ . 

 Por lo tanto, es fundamental investigar si los residentes, preceptores y tutores de la RMS están dispuestos a desarrollar la enseñanza, el aprendizaje y el cuidado, desde una perspectiva interprofesional ^(^ 11 ^)^ . Es deseable que estos profesionales desarrollen actividades colaborativas, conscientes de la importancia de compartir responsabilidades frente a las demandas de cuidado. Estudios nacionales e internacionales se han dedicado a medir la disponibilidad como forma de identificar problemas y promover el desarrollo de prácticas interprofesionales ^(^ 12 ^-^ 14 ^)^ . 

 La importancia de la educación interprofesional en entornos de aprendizaje clínico se basa en la oportunidad de desarrollar e integrar habilidades y competencias en el auténtico contexto sociocultural de los entornos de cuidados de salud en los que trabajarán los residentes ^(^ 5 ^)^ . Por lo tanto, medir la disponibilidad, incluso por medio de instrumentos autoinformados, puede proporcionar evidencia que respalde intervenciones personalizadas para promover habilidades colaborativas en el trabajo en equipo, con el fin de lograr objetivos comunes ^(^ 7 ^)^ . La forma en que los residentes perciben la práctica real puede diferir de los estereotipos o creencias que tenían antes de la experiencia colaborativa. Además, es posible que la ubicación de la práctica no coincida con su percepción de cómo sería una práctica interprofesional ^(^ 7 ^)^ . 

 Factores relacionados con la enseñanza pueden afectar la capacidad de formar profesionales colaborativos, como la socialización, el contexto de aprendizaje y el desarrollo del cuerpo docente. De esta manera, lo que se enseña en este contexto debe converger en términos de contenidos, es decir, las habilidades colaborativas deben estimular el reconocimiento de las responsabilidades de cada uno de los profesionales ^(^ 8 ^)^ . Una revisión sistemática, que tuvo como objetivo evaluar la efectividad de la educación interprofesional para profesionales y estudiantes de Enfermería y Medicina en cuanto a sus actitudes, habilidades, conocimientos y comportamientos, indicó que la evidencia sobre esta efectividad no es concluyente, demostrando la necesidad de otros estudios sobre el tema ^(^ 9 ^)^ . Asimismo, una revisión sistemática que tuvo como objetivo investigar los efectos de la educación interprofesional en la práctica colaborativa entre profesionales de la salud demostró que es un enfoque viable para mejorar las actitudes y el respeto mutuo entre estos profesionales, además de indicar que es necesario más investigación, considerando el desarrollo y la incorporación de la EIP en los planes de estudio, el bajo costo y los beneficios de este método de aprendizaje ^(^ 6 ^)^ . 

Esta investigación se basó en la siguiente pregunta de investigación: ¿cuál es el grado de disponibilidad de los residentes y de los preceptores para las prácticas interprofesionales y qué factores influyen en esa disposición? El objetivo del estudio fue evaluar el nivel de disponibilidad de residentes y de preceptores para las prácticas interprofesionales.

## Método

 Estudio de métodos mixtos del tipo triangulación concomitante, en el que se recogen datos cuantitativos y cualitativos de forma concomitante y luego se combinan. Este diseño de estudio mixto da el mismo peso a las dos etapas (CUAN + CUAL), ya que ambas tienen la misma relevancia para la consecución de los objetivos ^(^ 15 ^)^ . La combinación de datos se realizó por integración, proceso en el que elementos se fusionan y generan informaciones que se apoyan entre sí para comprender el objeto de estudio ^(^ 15 ^)^ . La elección de este enfoque fue influenciada por el conocimiento previo del programa de residencia, en el sentido de que éste se desarrolla en el hospital donde actúan los investigadores, reconociendo así las complejas interacciones entre residentes, preceptores, profesores y tutores. De este modo, al conocer la intención empírica de tener prácticas interprofesionales, manifestadas continuamente en clases y tutorías por todos los involucrados en los procesos de enseñanza y aprendizaje, se decidió atribuir el mismo peso a las etapas cuantitativa y cualitativa, pretendiendo investigar el fenómeno de manera concomitante. 

 La etapa cuantitativa se realizó a partir de un estudio observacional, de tipo descriptivo-analítico transversal, con recolección de datos basada en la aplicación de dos escalas, diseñado de acuerdo con el *Strengthening the Reporting of Observational Studies in Epidemiology* (STROBE). En la etapa cualitativa, se desarrolló una investigación exploratoria-descriptiva, con entrevistas, guiada por el *Consolidated Criteria for Reporting Qualitative Research* (COREQ). Para cumplir con el rigor metodológico del estudio mixto se utilizó la guía *Mixed Methods Appraisal Tool* (MMAT) ^(^ 16 ^)^ . 

### Escenario

 Estudio realizado en el programa de *Residência Integrada Multiprofissional em Saúde* (RIMS) de la *Universidade Federal de Santa Catarina* (UFSC), desarrollada en el *Hospital Universitário Polydoro Ernani de São Thiago* (HU-UFSC), ciudad de Florianópolis, Santa Catarina, Brasil. 

La RIMS fue creada en el HU-UFSC en 2010, a partir de una alianza entre los Departamentos de Enfermería, Farmacia, Fisioterapia, Fonoaudiología, Nutrición, Odontología, Psicología y Trabajo Social, con la dirección del HU. Los residentes son admitidos anualmente, con un proceso de selección que ofrece 36 vacantes en tres áreas de concentración: Atención de Urgencia y Emergencia, Atención de Salud de Alta Complejidad y Atención de Salud de la Mujer y el Niño.

### Población

Los participantes del estudio fueron residentes de primer y segundo año de todas las profesiones involucradas en la RIMS y preceptores que brindaban supervisión directa a los residentes. Es de destacar que los investigadores involucrados en este estudio no desempeñan el papel de preceptores en la residencia. Algunos de los investigadores actúan como tutores o profesores, y estos grupos no constituyeron la muestra. Cabe señalar también que estos últimos no participaron en la recolección de datos en ningún momento.

### Criterios de selección

Se adoptaron los siguientes criterios para participar en el estudio: 1) residentes: estar regularmente matriculados en el programa; y 2) preceptores: tener alguna vinculación (servidor o empleado público) con la institución hospitalaria y realizar actividad de supervisión directa con los residentes. Quedaron excluidos aquellos residentes y preceptores que se encontraban ausentes.

### Definición de la muestra y participantes

La definición de la muestra en la etapa cuantitativa se realizó utilizando diferentes estrategias para residentes y preceptores. En el caso de los residentes, el número de personas matriculadas en 2022 fue de 36 residentes de primer año (R1) y 27 residentes de segundo año (R2), totalizando 63 residentes. Para los preceptores, se calculó el promedio de profesionales en jornada de trabajo en un período de tres meses (febrero, marzo y abril de 2022). Esto se debió al alto ausentismo y rotación profesional. El cálculo inicial de la muestra indicó 204 profesionales elegibles y, tras los criterios de inclusión, quedó compuesta por 165 profesionales.

La etapa cualitativa del estudio estuvo compuesta por 26 participantes (entre los que participaron en la primera etapa). Las entrevistas fueron realizadas en un lugar privado dentro de la institución hospitalaria, por el investigador principal. El tiempo promedio de la entrevista fue de 27 minutos, con una duración que varió entre 18 y 38 minutos. Las entrevistas transcritas fueron enviadas individualmente a cada participante, para validar el contenido escrito, realizándose cambios cuando lo solicitara el entrevistado.

### Instrumentos utilizados

 Para la recolección de datos cuantitativos se utilizaron las escalas *Readiness for Interprofessional Learning Scale* (RIPLS) ^(^ 11 ^)^ y la Escala Jefferson de Actitudes Relacionadas con la Colaboración Interprofesional (EJARCI) ^(^ 17 ^)^ , ambas validadas para el portugués brasileño. 

 La RIPLS, que evalúa la disponibilidad de las personas para el desarrollo de la EIP, está compuesta por 27 ítems y se divide en tres factores: El Factor 1 se denomina Trabajo en Equipo y Colaboración y se relaciona con actitudes positivas hacia la disponibilidad para el aprendizaje compartido, la confianza y el respeto entre las áreas profesionales; el factor 2 se titula Identidad Profesional y se relaciona con aspectos específicos de las profesiones y la autonomía profesional; y el Factor 3 se denomina Atención de Salud Centrada en el Paciente, relacionado con actitudes positivas orientadas a las necesidades de salud desde la perspectiva del paciente. La puntuación general de la RIPLS varía entre 27 y 135 puntos, siendo 70, 40 y 25 la puntuación máxima para los Factores 1, 2 y 3, respectivamente ^(^ 11 ^)^ . 

 La escala EJARCI, adaptada y validada en Brasil en 2016, tiene como objetivo analizar actitudes relacionadas con la PC y está compuesta por 20 preguntas, siendo 140 la puntuación máxima ^(^ 17 ^)^ . En ambas escalas, la disponibilidad para EIP y PC está directamente relacionada con la puntuación, por lo que cuantos más puntos obtenga el encuestado, mayor será su disponibilidad para la EIP. Cabe destacar que RIPLS y EJARCI se complementaron con una sección dedicada a la caracterización de los participantes. 

Para recolectar datos cualitativos, las entrevistas fueron guiadas por un guión semiestructurado, con preguntas sobre cómo los participantes vivían las prácticas interprofesionales en su vida diaria, apertura al desarrollo, dificultades, potencialidades y aspectos sujetos a cambios. Este guion no fue validado, pero se realizó una prueba piloto con ex preceptores y ex residentes, que no constituyeron la muestra y cuyos datos no fueron incluidos en el estudio.

### Recolección de datos

Los datos cuantitativos y cualitativos fueron recolectados entre mayo de 2022 y febrero de 2023. Los datos cuantitativos fueron recolectados por una becaria contratada, estudiante de maestría de la misma institución, ajena a la RMS y quien fue capacitada en los instrumentos y cómo aplicarlos. Los participantes tuvieron la libertad de completar los formularios impresos al recibirlos o llevárselos a casa y devolverlos posteriormente. Luego de definida la muestra, se inició la distribución y aplicación de los instrumentos, simultáneamente con las entrevistas, las cuales fueron realizadas por el investigador principal.

 En la etapa cualitativa, para participar de las entrevistas, los residentes y preceptores fueron contactados vía correo electrónico y/o teléfono. Las invitaciones se realizaron de forma aleatoria, pero se observó representación en el número de preceptores y residentes en relación a las categorías profesionales. Para garantizar la imparcialidad, se utilizó una estrategia de aleatorización basada en criterios específicos, sin interferencia directa de los investigadores en la selección de los entrevistados. Esto se hizo manualmente, sin el uso de *software* de aleatorización, para garantizar una muestra representativa y variada. No hubo negativas a participar en las entrevistas. 

### Procesamiento y análisis de los datos

 Se utilizó el *software IBM SPSS Statistics 25.0* para analizar los datos cuantitativos. Se utilizó estadística descriptiva basada en medidas de tendencia central (media o mediana) y de dispersión (desviación estándar o rango intercuartil) para el análisis de los datos continuos, mientras que para los datos categóricos se utilizó el análisis de frecuencias absolutas y relativas. Para el análisis bivariado, la normalidad de los datos fue verificada mediante la prueba de Kolmogorov-Smirnov con ajuste de Lilliefors en todas las categorías (datos sociodemográficos y escalas RIPLS y EJARCI), indicando la adopción de la hipótesis alternativa de no normalidad (p < 0,05) para todas. Para las comparaciones entre participantes se utilizaron las pruebas no paramétricas U de Mann-Whitney para dos variables independientes y de Kruskal-Wallis para tres o más variables independientes, y, en esta última, cuando se rechazó H0, el análisis *post hoc* de Dunn. 

En cuanto a las escalas, cabe destacar que los ítems 10, 11, 12, 17, 19 y 21 de la RIPLS y los ítems 3, 5, 8, 9, 12, 15, 16 y 19 de la EJARCI se refieren a actitudes negativas relacionadas con EIP y PC y, por lo tanto, para el análisis de datos, las puntuaciones se invirtieron. Para determinar con precisión la disponibilidad de los participantes para EIP y PC, se decidió fragmentar la puntuación en cuartiles. De esta manera, cada 25% del puntaje global posible de alcanzar en ambas escalas correspondió a uno de los siguientes niveles de disponibilidad para el aprendizaje y la práctica interprofesional, que son: no disponible, baja disponibilidad, disponibilidad moderada y alta disponibilidad.

En cuanto a la edad, para efectos de análisis se dividió a los preceptores en un grupo de hasta 39 años y otro mayor de 39 años, considerando que la mediana de edad fue de 39 años. Para los residentes se consideró un grupo de 20 a 29 años y otro de 30 a 39 años, al no haber en la muestra residentes menores de 20 años y mayores de 39 años.

Para organizar los datos cualitativos, las entrevistas fueron grabadas en audio y transcritas en su totalidad. Para la confiabilidad de los datos cualitativos, tres de los investigadores de este estudio realizaron la discusión y la validación de los temas y subtemas surgidos de las declaraciones de los participantes, recopiladas a partir de entrevista semiestructurada. La opción de utilizar una metodología mixta también contribuyó para la confiabilidad de los datos cualitativos, ya que fue posible identificar la combinación de resultados cuantitativos obtenidos concomitantemente, ampliando continuamente la comprensión del fenómeno investigado.

 La información de las entrevistas fue analizada cualitativamente mediante el análisis de contenido temático, propuesto por Bardin ^(^ 18 ^)^ . El proceso analítico se llevó a cabo en tres fases: preanálisis, exploración del material y procesamiento de los datos. Se utilizó el software ATLAS.ti para las tres etapas del análisis de contenido, generándose 29 *codes* (unidades de registro) y 350 *quotations* (unidades de contexto). A partir de estas unidades se realizó el cruce de interpretaciones de los datos y la categorización temática. 

### Aspectos éticos

 El estudio fue aprobado por el Comité de Ética de la *Universidade Federal de Santa Catarina* bajo dictamen n.º 5.256.168. Se cumplieron todos los principios presentados por las Resoluciones n.º 466/2012 y n.º 510/2016 del *Conselho Nacional de Saúde* . Previamente, se explicó a los participantes todas las etapas de la investigación y se aclaró la participación opcional en cualquiera de ellas. Todos los participantes fueron informados y firmaron el Formulario de Consentimiento Informado. Para preservar la identidad, los participantes fueron identificados con la letra R de residente o P de preceptor y la sigla respectiva de la categoría profesional a la que pertenecían. Cuando había más de un participante de la misma profesión, se numeraban con números arábigos (1 o 2). 

## Resultados

 Participaron de la investigación 146 preceptores y 58 residentes. Se respetó el Índice de Confianza (IC) del 95% y un margen de error del 5%. En cuanto a los datos sociodemográficos, hubo predominio de participantes femeninos, tanto en el grupo de preceptores como en el grupo de residentes. La edad mínima de los participantes fue 22 años y la edad máxima fue 62 años. Las ocho profesiones incluidas en la RMS tuvieron representación similar en cuanto al número de participantes, con excepción de Enfermería, que tuvo el mayor número en comparación con todas las demás, por ser la profesión con mayor cantidad de plazas en el programa. En la Tabla 1 es posible observar el detalle de las características de los participantes. 

**Tabla 1:** - Características sociodemográficas de residentes y preceptores según frecuencia para variables categóricas y mediana para variables continuas. Florianópolis, SC, Brasil, 2023

**Variable**	N * (%) o mediana (mínimo-máximo)	
	**Preceptores** (N * =146)	**Residentes** (N * =58)
**Edad**	39 [27,0 – 62,0]	25 [22,0 – 35,0]
**Género**		
Masculino	19 (13,0%)	12 (20,7%)
Femenino	127 (87,0%)	46 (79,3%)
**Profesión**		
Enfermería	79 (54,1%)	16 (27,6%)
Fonoaudiología	9 (6,2%)	03 (5,2%)
Psicología	10 (6,8%)	11 (19,0%)
Farmacia	11 (7,5%)	7 (12,1%)
Trabajo Social	13 (8,9%)	11 (19,0%)
Fisioterapia	8 (5,5%)	2 (3,4%)
Nutrición	12 (8,2%)	5 (8,6%)
Odontología	4 (2,7%)	3 (5,2%)
**Área de concentración de la RMS ^†^**		
Alta Complejidad	93 (63,7%)	37 (63,8%)
Urgencia y Emergencia	13 (8,9%)	11 (19,0%)
Salud de la Mujer y el Niño	40 (27,4 %)	10 (17,2%)
**Tiempo desde la graduación**		
Hasta 2 años	NA ^‡^	26 (44,8%)
2 a 5 años	NA ^‡^	30 (51,7%)
Más de 5 años	NA ^‡^	2 (3,4%)
**Año de la RMS ^†^**		
Primer año (R1)	NA ^‡^	35 (60,3%)
Segundo año (R2)	NA ^‡^	23 (39,7%)
**Experiencia profesional**		
Hasta 2 años	1 (0,7%)	NA ^‡^
3 y 5 años	8 (5,5%)	NA ^‡^
6 y 10 años	34 (23,3%)	NA ^‡^
Más de 10 años	103 (70,5%)	NA ^‡^
**Tiempo trabajando como preceptor**		
Hasta 2 años	27 (18,5%)	NA ^‡^
Entre 3 y 5 años	53 (36,3%)	NA ^‡^
Entre 6 y 10 años	43 (29,5%)	NA ^‡^
Más de 10 años	22 (15,1%)	NA ^‡^
No respondió	1 (0,6%)	NA ^‡^

* N = Muestra

^†^ RMS = Residencia Multiprofesional en Salud

^‡^ NA = No aplicable

 En cuanto a la disponibilidad, todos los participantes se ubicaron en los percentiles de disponibilidad moderada y alta, tanto para la EIP, evaluada por la RIPLS, como para la PC, evaluada por la EJARCI. Para preceptores, la proporción en la RIPLS fue del 90,41% (132) con disponibilidad alta y del 9,58% (14) con disponibilidad moderada; en la EJARCI la proporción fue del 99,31% (145) con disponibilidad alta y 0,68% (1) con disponibilidad moderada. Para residentes, la proporción en la RIPLS fue del 94,82% (55) con disponibilidad alta y del 5,17% (3) con disponibilidad moderada; en la EJARCI, los 58 residentes lograron una alta disponibilidad. La Tabla 2 presenta la puntuación global de las dos escalas para residentes y preceptores. 

**Tabla 2:** - Puntuación de los Factores 1, 2 y 3 de la escala RIPLS* y puntuación global de las escalas RIPLS* y EJARCI **^†^** . Florianópolis, SC, Brasil, 2023

	Dimensiones RIPLS *			**Puntuación**	**Puntuación**
	**Fac 1** ^‡^	**Fac 2** ^§^	**Fac 3** ^||^	RIPLS *	**EJARCI** ^†^
**Preceptores (N** ¶ **=146)**					
Media	59,4	31,0	22,2	112,7	126,6
Desviación Estándar	5,5	2,9	2,2	8,2	8,4
Mediana	60,0	31,0	23,0	113,0	128,0
Puntuación mínima	43	22	16	89	100
Puntuación máxima	69	39	25	130	140
**Residentes (N** ¶ **=58)**					
Media	61,0	31,1	22,7	114,9	128,7
Desviación Estándar	4,0	2,9	2,2	6,9	7,6
Mediana	61,0	31,0	23,0	115,5	131,0
Puntuación mínima	51	23	17	95	108
Puntuación máxima	66	37	25	128	140

* RIPLS = *Readiness for Interprofessional Learning Scale*

^†^ EJARCI = Escala Jefferson de Actitudes Relacionadas con la Colaboración Interprofesional

^‡^ Fat 1 **=** Fator 1

^§^ Fat 2 = Fator 2

^||^ Fat 3 = Fator 3

^¶^ N = Muestra

Al comparar preceptores de diferentes profesiones, no hubo diferencias sugestivas en los puntajes de RIPLS y EJARCI, sin embargo, se observó una mediana más baja en el puntaje global de las dos escalas en enfermeros preceptores (RIPLS = 112 y EJARCI = 122). Lo mismo ocurrió cuando se realizaron pruebas comparando residentes de diferentes profesiones, sin embargo, las medianas más bajas observadas en este caso fueron en Trabajo Social para RIPLS (109) y Fonoaudiología para EJARCI (123).

 Específicamente para el tiempo transcurrido desde la graduación, hubo una diferencia sugestiva (P <0,014) para el Factor 1 de la RIPLS - Trabajo en Equipo y Colaboración. En la prueba *post hoc* se encontró diferencia entre residentes con hasta 2 años de egreso y entre aquellos con más de 5 años. 

 Al comparar puntuaciones globales entre preceptores y residentes de una misma profesión, los resultados indican diferencias entre dos profesiones, como se puede observar en la Tabla 3 . 

**Tabla 3:** - Comparación de la puntuación global en las escalas RIPLS* y EJARCI ^†^ según preceptores y residentes de la misma profesión. Florianópolis, SC, Brasil, 2023

	Dimensiones de la RIPLS*			**Puntuación**	
**Profesión**	**Fac 1** ^‡^	**Fac 2** §	**Fac 3** ^||^	RIPLS*	**EJARCI** ^†^
**ENF** ¶					
Preceptores	61,0 [45,0 – 67,0]	32,0 [24,0 – 39,0]	22,0 [16,0 – 25,0]	113,0 [96,0 – 130,0]	129,0 [100,0 – 140,0]
Residentes	60,0 [54,0 – 66,0]	32,0 [23,0 – 36,0]	22,5 [17,0 – 25,0]	118,0 [95,0 – 125,0]	131,5 [112,0 – 140,0]
*P* valor**	0,575	0,320	0,884	0,417	0,322
**FAM** ^††^					
Preceptores	60,0 [54,0 – 69,0]	31,0 [27,0 – 34,0]	22,0 [18,0 – 25,0]	111,0 [105,0 – 122,0]	126,0 [111,0 – 137,0]
Residentes	60,0 [51,0 – 66,0]	30,0 [26,0 – 34,0]	23,0 [18,0 – 25,0]	112,0 [101,0 – 125,0]	122,0 [108,0 – 134,0]
*P* valor**	0,930	0,596	0,479	0,930	0,536
**FSI** ^‡‡^	61,0 [56,0 – 66,0]	31,0 [28,0 – 37,0]	23,5 [20,0 – 25,0]	117,5 [107,0 – 123,0]	130,0 [110,0 – 140,0]
Preceptores					
Residentes	64,5 [63,0 – 66,0]	32,0 [30,0 – 34,0]	22,5 [20,0 – 25,0]	119,0 [113,0 – 125,0]	129,0 [124,0 – 134,0]
*P* valor**	0,400	0,711	1,000	0,533	1,000
**FON** ^§§^					
Preceptores	58,0 [51,0 – 66,0]	31,0 [27,0 – 33,0]	23,0 [20,0 – 25,0]	112,0 [106,0 – 122,0]	124,0 [120,0 – 137,0]
Residentes	64,0 [64,0 – 65,0]	35,0 [29,0 – 35,0]	24,0 [23,0 – 24,0]	122,0 [117,0 – 124,0]	134,0 [133,0 – 139,0]
*P* valor**	0,064	0,373	0,373	**0,018**	**0,036**
**NUT** ^||||^	56,0 [43,0 – 66,0]	29,5 [27,0 – 25,0]	22,5 [19,0 – 25,0]	110,0 [89,0 – 119,0]	123,0 [111,0 – 135,0]
Preceptores					
Residentes	64,0 [59,0 – 66,0]	31,0 [29,0 – 35,0]	25,0 [21,0 – 25,0]	120,0 [116,0 – 123,0]	133,0 [122,0 -140,0]
*P* valor**	**0,004**	0,442	0,082	**0,001**	**0,037**
**ODO** ^¶¶^					
Preceptores	60,5 [58,0 – 66,0]	31,0 [29,0 – 33,0]	21,5 [20,0 – 23,0]	112,5 [109,0 – 121,0]	124,0 [118,0 – 131,0]
Residentes	60,0 [55,0 – 61,0]	32,0 [30,0 – 34,0]	25,0 [20,0 – 25,0]	114,0 [110,0 – 118,0]	139,0 [133,0 – 140,0]
*P* valor**	0,629	0,629	0,400	0,857	0,057
PSI***					
Preceptores	63,0 [49,0 – 66,0]	32,5 [29,0 – 35,0]	24,0 [21,0 – 25,0]	119,0 [103,0 – 123,0]	127,0 [116,0 – 138,0]
Residentes	61,0 [57,0 – 66,0]	31,0 [27,0 – 37,0]	23,0 [20,0 – 25,0]	113,0 [109,0 – 128,0]	126,0 [117,0 – 135,0]
*P* valor**	0,173	0,314	0,468	0,223	0,756
**TSO** ^†††^					
Preceptores	60,00 [49,0 – 66,0]	28,0 [22,0 – 35,0]	23,0 [20,0 – 25,0]	109,0 [98,0 – 125,0]	125,0 [109,0 – 140,0]
Residentes	60,0 [55,0 – 66,0]	28,0 [26,0 – 33,0]	23,0 [19,0 – 25,0]	115,0 [100,0 – 124,0]	130,0 [111,0 – 139,0]
*P* valor**	0,733	0,733	0,820	0,361	0,569

* RIPLS = *Readiness for Interprofessional Learning Scale*

^†^ EJARCI = Escala Jefferson de Actitudes Relacionadas con la Colaboración Interprofesional

^‡^ Fat 1 **=** Fator 1

^§^ Fat 2 = Fator 2

^||^ Fat 3 = Fator 3

^¶^ ENF = Enfermería

** Prueba U de Mann-Whitney

^††^ FAM = Farmacia

^‡‡^ FIS = Fisioterapia

^§§^ FON = Fonoaudiología

^||||^ NUT = Nutrición

^¶¶^ ODO = Odontología

*** PSI = Psicología

^†††^ SSO = Trabajo Social

La hipótesis nula adoptada fue que la distribución de puntuaciones sería igual entre residentes y preceptores. Esta hipótesis fue rechazada para Fonoaudiología en la puntuación global de la RIPLS (U= 1,500; p < 0,018) y en la puntuación global de la EJARCI (U= 2,500; p < 0,036). También fue rechazada para Nutrición en la puntuación del Factor 1 de la RIPLS (U= 4,500; p < 0,004), puntuación global de la RIPLS (U= 2,500; p < 0,001) y puntuación global de la EJARCI (U= 10,500; p < 0,037).

El análisis temático indicó que el proceso de formación de pregrado y la resistencia de algunos profesionales son dos de las principales oportunidades para mejorar la disponibilidad. Se destacaron como principales potencialidades la voluntad de diálogo horizontal y la colaboración del equipo multiprofesional, tanto entre preceptores como residentes.

La formación de profesionales, mayoritariamente desde una perspectiva uniprofesional, juega un papel limitante para las prácticas interprofesionales, debido a la falta de reconocimiento de los roles de compañeros de otra categoría profesional, como se puede comprobar en las declaraciones.

*Todavía estamos avanzando hacia esto* [las prácticas interprofesionales] *, no es algo específico de aquí, pero también resulta de nuestra formación, que solamente está enfocada a nuestra categoría profesional, no tenemos conocimiento de la parte técnica de otros profesionales para que tomen las acciones que vemos que toman.* (P.FON) 

*Los propios docentes tienen dificultad para poner nuestra profesión en las clases en un contexto interprofesional. Incluso lo entiendo, porque si bien todos somos profesiones de la salud, nos capacitamos por separado y hay diferentes segmentos dentro de cada profesión, entonces entiendo la dificultad.* (R.ODO1) 

*Tengo dificultades, sobre todo en las clases, porque no tengo formación clínica, no vemos nada del cuerpo* [anatomía, fisiología, etc.] *, nuestra formación está aislada de la de todos los demás profesionales de la salud. Así que a veces ni siquiera entiendo de qué están hablando, la mayoría de las cosas tengo que preguntar de qué se trata.* (R.TSO1) 

*Tenemos dificultades para relacionarnos con otros profesionales, ya que está muy restringido a nuestra área. En Odontología lamentablemente contamos con una formación que no involucra a otros profesionales, por lo que tenemos la dificultad de saber trabajar con todos para satisfacer las necesidades de los pacientes.* (R.ODO2) 

*Veo que terminamos intentando explicar a qué vinimos todo el tiempo. Veo más bien que la Psicología y el Trabajo Social realmente necesitan demostrar por qué están ahí, la gente tiene dificultades para demandar el Trabajo Social o la Psicología, en gran parte por su formación universitaria también, que está más aislada de otras profesiones.* (R.PSI1) 

En cuanto al perfil profesional, las influencias se dan por la resistencia de algunos profesionales a desarrollar la colaboración tanto para la docencia como para el trabajo. Existe un temor a compartir información y una tendencia a actuar de forma autónoma, principalmente cuando se trata de estudiantes o residentes.

*Hay mucha resistencia por parte del personal, es muy... no sé si esa es la palabra correcta, pero egoísmo. Hay algunas profesiones en las que se nota que no quieren compartir conocimientos porque tienen miedo de que otros sepan más que ellos, esto nos desanima, ¿cómo vamos a aprender juntos así?* (R.FAM1) 

*Hay un compañero enfermero que decía “no me gustan los estudiantes, no quiero a nadie de la residencia conmigo”. Estos profesionales hacían las cosas sin los estudiantes, para hacerlo más rápido, lo que terminaba dejándolos sin ganas de aprender.* (P.ENF1) 

*Vemos quién está de turno, buscamos a una persona que ya conocemos, que sabemos que tiene una actitud más amable, más sensibilidad, más colaborativa. A veces prefiero no hablar del caso dependiendo de quién sea el otro profesional, porque sé que no progresará, no me escuchará.* (P.TSO) 

Por otro lado, el acceso facilitado y el diálogo horizontal influyen en el deseo de aprender a trabajar de modo colaborativo en la disponibilidad. El diálogo efectivo y la paridad en el peso de las contribuciones dentro del equipo multiprofesional respaldan el deseo de actuar colaborativamente.

*De ahí la importancia de tener un buen acceso al equipo. En la UCI NEO puedo hablar con el médico todo el tiempo, con los enfermeros. El equipo tiene un sistema de reuniones para discutir casos y conductas y no solamente para discutir conductas, sino para discutir cambios.* (P.TSO) 

*Aquí sentí que podíamos comunicarnos con el equipo y establecer objetivos juntos. El equipo multiprofesional de rehabilitación, fisio, fono, nutri, psicólogo y enfermería, lo que nos motiva a querer compartir ideas.* (P.FIS1)**

*Luego, médicos, enfermeros y otros profesionales acuden a mí para hacerme preguntas e intercambiar ideas. Es muy abierto y tranquilo, es un buen diálogo.* (R.FIS) 

*En la UCI NEO cada caso se discute intensamente con todas las profesiones, hay un desarrollo conjunto, no se toma ninguna decisión sin ser discutida con la fono, enfermería, trabajo social. Se habla mucho de todo. Vemos como el otro es importante.* (R.ENF2) 

*Hay personas más dispuestas, más accesibles, más fáciles de tratar, y con esas personas el trabajo fluye.* (P.ENF1) 

La formación de pregrado influye en el desarrollo de las prácticas interprofesionales, en las perspectivas de los residentes y de los preceptores de Trabajo Social y Psicología, quienes relataron dificultades para trabajar en el equipo multiprofesional, debido a las limitaciones en la comprensión de los aspectos biológicos del cuidado y la falta de reconocimiento de la importancia de los aspectos psicosociales por parte de otras profesiones, como la Odontología, cuya formación está dirigida a los aspectos anatómicos y fisiopatológicos de la boca, y no hay tanta predisposición al diálogo con estudiantes de otras profesiones del área de la salud.

 Para la integración y la comprensión de los datos, se utilizó la técnica de *joint-display* , que se muestra en la Figura 1 . 

**Figura 1:** - Joint-display para la integración de los datos cuantitativos y cualitativos. Florianópolis, SC, Brasil, 2023

*M _e_ = Media; ^†^ EIP = Educación Interprofesional; ^‡^ PC = Práctica Colaborativa; §Prueba U de Mann-Whitney; ^||^ FON = Fonoaudiología; ¶NUT = Nutrición; **Mayor frecuencia de respuestas; ^††^ Prueba de Kruskal-Wallis

## Discusión

 Las prácticas interprofesionales juegan un papel importante a la hora de fomentar el intercambio de experiencias entre equipos multiprofesionales y entre residentes de diferentes profesiones, promoviendo la centralidad del cuidado al paciente y la convergencia de áreas de conocimiento ^(^ 1 ^-^ 3 ^)^ . En el contexto de este estudio, los hallazgos indican alta disponibilidad de los participantes para el desarrollo de las prácticas interprofesionales en los ámbitos educativos y de cuidado. 

 Cabe señalar que esta alta disponibilidad puede estar relacionada con el contexto en el que se encuentran los participantes, sobre todo teniendo en cuenta el entorno de los hospitales universitarios, que son favorables a la aplicación de innovaciones en la prestación de cuidados, debido a sus conexiones con universidades, lo que fomenta la enseñanza y la investigación ^(^ 19 ^)^ . Además, la RMS ofrece un enfoque diferenciado a los estudios de posgrado, permitiendo a residentes y preceptores la oportunidad de aprendizaje y práctica simultáneos, e incentivando la colaboración entre profesionales de diferentes áreas ^(^ 8 ^-^ 9 ^,^ 19 ^)^ . De este modo, se puede intensificar la conciencia de la importancia de la colaboración en la enseñanza y en el cuidado, promoviendo la disposición para las prácticas interprofesionales. 

 A pesar de esto, los participantes indican que la voluntad por sí sola no es suficiente para implementar prácticas interprofesionales consistentes en la RMS hospitalaria. Esto puede estar relacionado con las rutinas hospitalarias, que aún permanecen rígidas, con roles profesionales delimitados por fronteras históricamente definidas, a menudo marcadas por la falta de autonomía o por responsabilidades relacionadas con la ejecución técnica de los procedimientos. El predominio del modelo biomédico aún sitúa el diagnóstico y la curación como foco, resultando en la sobrevaloración de las prácticas médicas y en una menor autonomía y reconocimiento de otros profesionales, lo que impacta negativamente la valoración del trabajo colaborativo ^(^ 19 ^)^ . 

 El modelo biomédico mantiene su influencia histórica en el área de la salud, no solamente en los cuidados clínicos, sino también en la formación. Este estudio destaca que la formación de pregrado, salvo algunas iniciativas aisladas, muchas veces mantiene un enfoque disciplinario y uniprofesional del conocimiento, lo que puede limitar las oportunidades de los estudiantes de aprender unos de otros y entre ellos ^(^ 6 ^)^ . La falta de conciencia sobre la colaboración en los cursos de pregrado dificulta la formación de profesionales colaborativos. Además, esta brecha puede perpetuar un ciclo de resistencia o adopción parcial de prácticas interprofesionales, especialmente en la medida en que estos estudiantes tengan dificultades para integrar conocimientos y acciones interprofesionales en futuros lugares de trabajo ^(^ 20 ^)^ . 

 Sin embargo, atribuir parte de las dificultades del trabajo interprofesional a la formación de pregrado puede enmascarar las dificultades para comprender los límites establecidos dentro de cada área profesional, que parecen estar más definidos en Medicina, en comparación con otras profesiones ^(^ 21 ^)^ . La idea de necesitar aprender la profesión de cada uno para luego contribuir de manera interprofesional, entra en conflicto con la necesidad de que múltiples profesionales en su propia formación trabajen juntos, cada uno en su propio ámbito, y luego colaboren entre sí ^(^ 5 ^)^ . Otro punto crítico para el desarrollo de la EIP y de la PC, según indican los participantes en este estudio, son ciertas características profesionales, especialmente con relación a la comunicación, herramienta esencial en el área de la salud ^(^ 2 ^,^ 22 ^)^ . La comunicación ineficaz y la falta de apertura al diálogo interprofesional comprometen la enseñanza y el cuidado, perpetuando la fragmentación de las actividades asistenciales y del conocimiento. 

 Considerando un enfoque de la práctica interprofesional basado en competencias, la comunicación emerge como un atributo fundamental ^(^ 23 ^-^ 24 ^)^ . La comunicación eficaz, empática y no violenta no solamente facilita la colaboración interprofesional, sino que también crea entornos de aprendizaje saludables y prácticas de cuidados seguros, minimizando errores y promoviendo el cuidado ^(^ 24 ^)^ . En este contexto, las habilidades comunicativas requieren capacitación y perfeccionamiento, permitiendo cambios positivos en las actitudes y comportamientos de los profesionales e incentivando un diálogo constante y acogedor dentro de la RMS. 

Este estudio contribuye significativamente al avance del conocimiento en el área de la salud, abordando un tema de relevancia mundial. Para implementar prácticas interprofesionales es fundamental, entre otras acciones, medir la disponibilidad y comprender los factores que influyen en la adhesión de profesionales y estudiantes a este enfoque. La RMS aparece como una herramienta prometedora para la incorporación de estas prácticas en el contexto hospitalario brasileño, justificando plenamente su investigación.

Entre las limitaciones de este estudio se destaca la ausencia de profesores y tutores de la RMS como participantes, así como de pacientes y familiares, cuya inclusión podría enriquecer la comprensión del fenómeno, especialmente en el ámbito cualitativo. Además, la inclusión de profesionales y residentes de Medicina proporcionaría una visión integral de las dinámicas interprofesionales y su impacto en la disposición a colaborar, ya que evaluar a los profesionales de la residencia multiprofesional sin considerar el papel del médico en el abordaje del paciente indica un sesgo de selección, ya que, después de todo, las acciones del equipo multiprofesional incluyen enfoques de todas las profesiones del área de la salud. Asimismo, los residentes y preceptores con actitudes más positivas hacia la EIP pueden haber tenido más probabilidades de participar en el estudio, lo que puede haber influido en los resultados.

## Conclusión

El estudio presentó la disponibilidad para el desarrollo de prácticas interprofesionales de residentes y preceptores de una Residencia Multiprofesional en Salud hospitalaria. Los resultados cuantitativos indicaron una alta disponibilidad para la Educación Interprofesional y la Práctica Colaborativa entre los participantes y, a partir de la integración de los datos recolectados concomitantemente, los resultados cualitativos permitieron comprender los factores involucrados en esta disponibilidad para la EIP y la PC.

Pese a ello, la dificultad para desarrollar y mantener prácticas interprofesionales incluye la formación de pregrado y las características de los profesionales involucrados, como las dificultades en la comunicación entre profesiones y la apertura al diálogo. Estas dificultades pueden y deben observarse desde la perspectiva de las oportunidades, buscando perfeccionar las habilidades comunicativas de residentes, preceptores y profesionales en general, con miras a mejorar los procesos de enseñanza y de trabajo en el ámbito de las Residencias Multiprofesionales en Salud.

# Translation: Disponibilidade de residentes e preceptores para práticas interprofissionais: estudo de métodos mistos *

**DOI:** 10.1590/1518-8345.7374.4396

**Published:** 2024-10-03

**Authors:** André Lucas Maffissoni, Jussara Gue Martini, Daniele Delacanal Lazzari, Carine Vendruscolo, Marina da Silva Sanes, Paula Bresolin

**Affiliations:** ^1^ Universidade Federal de Santa Catarina, Florianópolis, SC, Brasil; ^2^ Bolsista do Conselho Nacional de Desenvolvimento Científico e Tecnológico (CNPq), Brasil.; ^3^ Universidade do Estado de Santa Catarina, Departamento de Enfermagem, Chapecó, SC, Brasil.

**Keywords:** Residência em Saúde, Educação Interprofissional, Prática Interdisciplinar, Relações Interprofissionais, Comunicação Interdisciplinar, Educação em Saúde

## Abstract

avaliar o nível de disponibilidade de residentes e de preceptores para as práticas interprofissionais.

estudo de métodos mistos do tipo triangulação concomitante, realizado com residentes e preceptores de uma Residência Multiprofissional em Saúde hospitalar.

mais de 90% (n=186) dos participantes possuem alta disponibilidade para as práticas interprofissionais. Contudo, práticas instituídas durante a graduação e o perfil profissional dos envolvidos, especialmente no que diz respeito à falta de comunicação entre as profissões e a abertura ao diálogo, foram indicados como dificuldades para a efetivação da interprofissionalidade no cenário investigado, o que pode influenciar negativamente a disponibilidade de residentes e preceptores.

apesar de residentes e preceptores possuírem alta disponibilidade para o desenvolvimento da interprofissionalidade, determinados fatores dificultam sua execução, obstando o trabalho colaborativo.

## Destaques

**(1)** O nível de disponibilidade influencia as práticas interprofissionais hospitalares.

**(2)** Dificuldades de comunicação interprofissional impedem práticas colaborativas.

**(3)** Há resistência na adoção de práticas interprofissionais relacionadas à formação.

**(4)** Residências são espaços importantes para desenvolver práticas interprofissionais.

## Introdução

 As práticas interprofissionais em saúde englobam um conjunto de ações executadas por equipes multiprofissionais, visando a elaboração e a execução coletivas de cuidados qualificados ^(^ 1 ^)^ . Diante da complexidade inerente aos cenários hospitalares, tanto em âmbito nacional quanto internacional, são propostas diversas estratégias destinadas a incentivarem esse processo colaborativo, tais como a Educação Interprofissional (EIP) e a Prática Colaborativa (PC). 

 A EIP materializa-se por meio da coaprendizagem de estudantes oriundos de distintas áreas, visando à amplificação da colaboração e, por consequência, a otimização dos desfechos na saúde. A PC, por outro lado, pretende a convergência de profissionais da saúde com expertise variada, envolvendo pacientes, familiares, cuidadores e comunidades, com o intuito de gerar cuidados voltados à segurança do paciente ^(^ 1 ^-^ 2 ^)^ . Embora possuam diferenças conceituais, essas estratégias revelam-se interdependentes, uma vez que a colaboração no trabalho é impulsionada pelo aprendizado mútuo, e, inversamente, a aprendizagem colaborativa desenvolve-se em uma perspectiva unificada de atuação. 

 As práticas colaborativas podem ser fomentadas por meio de disciplinas curriculares ou mesmo de propostas a serem operacionalizadas nas diferentes ênfases, tais como encontros específicos com temas comuns, tutorias ou outras atividades deliberadas que, a partir do pensamento crítico, estabeleçam estratégias a serem discutidas e ações em conjunto para cada paciente ^(^ 3 ^)^ . Todavia, essa colaboração não ocorre sem obstáculos, especialmente no contexto hospitalar, uma vez que, apesar dos avanços na prestação de cuidados nesses cenários, persiste uma tendência ao cuidado uniprofissional, centrado no modelo biomédico, frequentemente ancorado na patologia ^(^ 4 ^-^ 6 ^)^ . 

 A Residência Multiprofissional em Saúde (RMS) hospitalar foi iniciada no ano de 2010, com o objetivo de contribuir para a formação de profissionais para o Sistema Único de Saúde (SUS), traçando novas possibilidades de aprendizagem e trabalho, em um espaço historicamente marcado pela fragmentação do cuidado. Os méritos da RMS na melhoria dos cuidados hospitalares são inegáveis, embora persistam dificuldades para concretizar estratégias relacionadas às PC ^(^ 7 ^-^ 10 ^)^ . 

 Desta forma, é fundamental investigar se residentes, preceptores e tutores da RMS estão dispostos a desenvolverem o ensino, a aprendizagem e o cuidado, pautados em uma perspectiva interprofissional ^(^ 11 ^)^ . É desejável que esses profissionais desenvolvam atividades colaborativas, sensibilizados da importância do compartilhamento de responsabilidades acerca das demandas de cuidado. Estudos nacionais e internacionais têm se dedicado à mensuração da disponibilidade como uma maneira de identificar problemas e promover o desenvolvimento das práticas interprofissionais ^(^ 12 ^-^ 14 ^)^ . 

 A importância da educação interprofissional em ambientes de aprendizagem clínica está alicerçada na oportunidade de desenvolver e integrar habilidades e competências no autêntico contexto sociocultural dos ambientes de cuidados de saúde nos quais os residentes trabalharão ^(^ 5 ^)^ . Assim, mensurar a disponibilidade, mesmo que por meio de instrumentos autorrelatados, pode fornecer evidências que apoiem intervenções personalizadas para promover competências colaborativas no trabalho em equipe, a fim de alcançar objetivos comuns ^(^ 7 ^)^ . A forma como os residentes percebem a prática real pode diferir de estereótipos ou crenças que possuíam antes da experiência colaborativa. Além disso, o local de prática pode não corresponder à sua percepção sobre o que seria uma prática interprofissional ^(^ 7 ^)^ . 

 Fatores relacionados ao ensino podem afetar a capacidade de formação de profissionais colaborativos, tais como a socialização, o contexto de aprendizagem e o desenvolvimento do corpo docente. Desta forma, o que é ensinado neste contexto deve convergir em termos de conteúdo, ou seja, competências colaborativas devem estimular o reconhecimento das atribuições de cada um dos profissionais ^(^ 8 ^)^ . Uma revisão sistemática, que possuía como objetivo avaliar a eficácia da educação interprofissional para profissionais e estudantes de Enfermagem e Medicina sobre suas atitudes, habilidades, conhecimentos e comportamentos, indicou que as evidências sobre esta eficácia são inconclusivas, demonstrando a necessidade de outros estudos sobre o tema ^(^ 9 ^)^ . Já uma revisão sistemática que teve como objetivo investigar os efeitos da educação interprofissional na prática colaborativa entre profissionais de saúde, demonstrou que é uma abordagem viável para melhorar atitudes e respeito mútuo entre estes profissionais, bem como indicou que outras pesquisas são necessárias, considerando o desenvolvimento e a incorporação da EIP nos currículos, o baixo custo e os benefícios deste método de aprendizagem ^(^ 6 ^)^ . 

Esta investigação partiu da seguinte questão de pesquisa: qual o grau de disponibilidade de residentes e preceptores para as práticas interprofissionais, e quais fatores influenciam essa disposição? O objetivo do estudo foi avaliar o nível de disponibilidade de residentes e de preceptores para as práticas interprofissionais.

## Método

 Estudo de métodos mistos do tipo triangulação concomitante, em que os dados quantitativos e qualitativos são coletados concomitantemente e depois mixados. Este *design* de estudo misto atribui o mesmo peso para as duas etapas (QUAN + QUAL), dado que ambas possuem a mesma relevância para o alcance dos objetivos ^(^ 15 ^)^ . A mixagem de dados foi realizada por integração, processo no qual os elementos são fundidos e geram informações que se apoiam mutuamente para a compreensão do objeto do estudo ^(^ 15 ^)^ . A escolha dessa abordagem foi influenciada pelo conhecimento prévio do programa de residência, no sentido de que este é desenvolvido no hospital em que os pesquisadores trabalham, reconhecendo, portanto, as interações complexas entre residentes, preceptores, professores e tutores. Desta forma, ao saber da intencionalidade empírica de haver práticas interprofissionais, manifestadas continuamente em aulas e tutorias por todos os envolvidos nos processos de ensino e aprendizagem, optou-se por atribuir o mesmo peso às etapas quanti e qualitativas, intencionando investigar o fenômeno de maneira concomitante. 

 A etapa quantitativa foi realizada a partir de um estudo observacional, do tipo transversal descritivo-analítico, com coleta de dados a partir da aplicação de duas escalas, delineado de acordo com o *Strengthening the Reporting of Observational Studies in Epidemiology* (STROBE). Na etapa qualitativa, foi desenvolvida uma pesquisa exploratório-descritiva, com entrevistas, norteada pelo *Consolidated Criteria for Reporting Qualitative Research* (COREQ). Para cumprimento do rigor metodológico do estudo misto, utilizou-se o guia *Mixed Methods Appraisal Tool* (MMAT) ^(^ 16 ^)^ . 

### Cenário

Estudo realizado no programa de Residência Integrada Multiprofissional em Saúde (RIMS) da Universidade Federal de Santa Catarina (UFSC), desenvolvida no Hospital Universitário Polydoro Ernani de São Thiago (HU-UFSC), município de Florianópolis, Santa Catarina, Brasil.

A RIMS foi criada no HU-UFSC no ano de 2010, a partir de parceria entre os Departamentos de Enfermagem, Farmácia, Fisioterapia, Fonoaudiologia, Nutrição, Odontologia, Psicologia e Serviço Social, com a diretoria do HU. O ingresso de residentes ocorre anualmente, com um processo seletivo que dispõe de 36 vagas em três áreas de concentração: Atenção em Urgência e Emergência, Atenção em Alta Complexidade em Saúde e Atenção à Saúde da Mulher e da Criança.

### População

Os participantes do estudo foram residentes do primeiro e segundo anos, de todas as profissões envolvidas na RIMS, e preceptores que realizavam supervisão direta aos residentes. Destaca-se que os pesquisadores envolvidos neste estudo não exercem o papel de preceptores na residência. Parte dos pesquisadores atua como tutor ou professor, e estes grupos não compuseram a amostra. Ressalta-se, ainda, que estes últimos não participaram da coleta de dados em nenhum momento.

### Critérios de seleção

Como critérios para participar do estudo foram adotados: 1) residentes: estar regularmente matriculado no programa; e 2) preceptores: possuir qualquer vínculo (servidor ou empregado público) com a instituição hospitalar e exercer atividade de supervisão direta com os residentes. Excluíram-se aqueles residentes e preceptores que estavam afastados.

### Definição da amostra e participantes

A definição da amostra na etapa quantitativa foi realizada por estratégias diferentes para residentes e preceptores. No caso dos residentes, o quantitativo de matriculados no ano de 2022 era de 36 residentes do primeiro ano (R1) e 27 residentes do segundo ano (R2), totalizando 63 residentes. Para os preceptores, calculou-se a média de profissionais em escala de trabalho em um período de três meses (fevereiro, março e abril de 2022). Isto se deu em função do alto absenteísmo e da rotatividade dos profissionais. O cálculo inicial da amostra indicou 204 profissionais elegíveis, e, após os critérios de inclusão, constitui-se em 165 profissionais.

A etapa qualitativa do estudo foi composta por 26 participantes (dentre aqueles que participaram da primeira etapa). As entrevistas foram realizadas em local reservado dentro da instituição hospitalar, pelo pesquisador principal. O tempo médio das entrevistas foi de 27 minutos, com amplitude variando entre 18 e 38 minutos. As entrevistas transcritas foram encaminhadas individualmente para cada participante, para validação do conteúdo escrito, fazendo-se alterações quando solicitado pelo entrevistado.

### Instrumentos utilizados

 Para a coleta dos dados quantitativos foram utilizadas as escalas *Readiness for Interprofessional Learning Scale* (RIPLS) ^(^ 11 ^)^ e Escala Jefferson de Atitudes Relacionadas à Colaboração Interprofissional (EJARCI) ^(^ 17 ^)^ , ambas validadas para o português brasileiro. 

 A RIPLS, que avalia a disponibilidade dos sujeitos para o desenvolvimento da EIP, é composta por 27 itens e dividida em três fatores: O Fator 1 é denominado de Trabalho em Equipe e Colaboração e está relacionado com atitudes positivas à disponibilidade para aprendizagem compartilhada, confiança e respeito entre as áreas profissionais; o Fator 2 é titulado como Identidade Profissional e se relaciona com aspectos específicos das profissões e autonomia profissional; e o Fator 3 é nominado como Atenção à Saúde Centrada no Paciente, relacionando-se com atitudes positivas direcionadas às necessidades de saúde na ótica do paciente. A pontuação global da RIPLS varia entre 27 e 135 pontos, sendo 70, 40 e 25 a pontuação máxima para os Fatores 1, 2 e 3, respectivamente ^(^ 11 ^)^ . 

 A escala EJARCI, adaptada e validada no Brasil no ano de 2016, é voltada para a análise das atitudes relacionadas à PC, e é composta por 20 questões, sendo 140 a pontuação máxima ^(^ 17 ^)^ . Nas duas escalas, a disponibilidade para a EIP e a PC está diretamente relacionada à pontuação, assim, quanto mais pontos o respondente obtiver, maior sua disponibilidade para a EIP. Ressalta-se que a RIPLS e a EJARCI foram complementadas com uma seção destinada à caracterização dos participantes. 

Para a coleta de dados qualitativos, as entrevistas foram guiadas por roteiro semiestruturado, com perguntas sobre como os participantes vivenciavam as práticas interprofissionais no seu cotidiano, a abertura para o desenvolvimento, as dificuldades, as potencialidades e os aspectos passíveis de mudanças. Este roteiro não foi validado, mas realizou-se teste piloto com ex-preceptores e ex-residentes, que não compuseram a amostra e cujos dados não integraram o estudo.

### Coleta de dados

Os dados quantitativos e qualitativos foram coletados entre maio de 2022 e fevereiro de 2023. Os dados quantitativos foram coletados por bolsista contratada, mestranda na mesma instituição, sem relação com a RMS e que foi treinada sobre os instrumentos e como aplicá-los. Os participantes tiveram a liberdade de preencher os formulários impressos no momento de recebimento ou levá-los para casa e devolvê-los posteriormente. Após a definição da amostra, iniciou-se a distribuição e aplicação dos instrumentos, em concomitância com a realização das entrevistas, que foram feitas pelo pesquisador principal.

 Na etapa qualitativa, para participação nas entrevistas, os residentes e preceptores foram contatados por e-mail e/ou telefone. Os convites foram realizados de forma aleatória, mas foi observada a representatividade no número de preceptores e residentes com relação às categorias profissionais. Para garantir a imparcialidade, utilizou-se estratégia de aleatorização baseada em critérios específicos, sem interferência direta dos pesquisadores na seleção dos entrevistados. Isso foi feito de forma manual, sem o uso de *software* aleatorizador, para assegurar uma amostra representativa e variada. Não houve negativas para participação nas entrevistas. 

### Tratamento e análise dos dados

 Para a análise dos dados quantitativos foi utilizado o *software IBM SPSS Statistics 25.0* . Utilizou-se a estatística descritiva a partir de medidas de tendência central (média ou mediana) e de dispersão (desvio padrão ou amplitude interquartil) para a análise dos dados contínuos, enquanto a análise de frequências absolutas e relativas foi utilizada para os dados categóricos. Para a análise bivariada, a verificação da normalidade dos dados foi realizada por meio do teste Kolmogorov-Smirnov com ajuste de Lilliefors sobre todas as categorias (dados sociodemográficos e escalas RIPLS e EJARCI), indicando a adoção da hipótese alternativa de não normalidade (p < 0,05) para todas. Para comparações entre os participantes foram utilizados os testes não paramétricos U de Mann-Whitney para duas variáveis independentes e Kruskal-Wallis para três ou mais variáveis independentes, e, neste último, quando houve rejeição da H0, a análise de *post hoc* de Dunn. 

Com relação às escalas, ressalta-se que os itens 10, 11, 12, 17, 19 e 21 da RIPLS e os itens 3, 5, 8, 9, 12, 15, 16 e 19 da EJARCI se referem a atitudes negativas relacionadas à EIP e à PC, e, diante disso, para a análise dos dados, os escores foram invertidos. Para determinar com precisão a disponibilidade dos participantes para a EIP e a PC, optou-se por fragmentar a pontuação em quartis. Desta forma, cada 25% da pontuação global possível de se alcançar nas duas escalas correspondeu a um dos seguintes níveis de disponibilidade para o aprendizado e para a prática interprofissional, que são: não disponível, baixa disponibilidade, moderada disponibilidade e alta disponibilidade.

Com relação à idade, para fins de análise, os preceptores foram divididos em um grupo de até 39 anos e em outro acima de 39 anos, considerando-se que a mediana de idade foi de 39 anos. Para os residentes, foram considerados um grupo entre 20 e 29 anos e outro entre 30 e 39 anos, em virtude de não haver residentes abaixo de 20 anos e acima de 39 anos na amostra.

Para a organização dos dados qualitativos, as entrevistas foram áudio-gravadas e transcritas na íntegra. Para a confiabilidade dos dados qualitativos, três dos pesquisadores deste estudo realizaram a discussão e a validação dos temas e subtemas oriundos dos depoimentos dos participantes, coletados a partir de entrevista semiestruturada. A opção por utilizar uma metodologia mista também contribuiu para a confiabilidade dos dados qualitativos, pois foi possível identificar a mixagem dos resultados quantitativos obtidos concomitantemente, continuamente ampliando a compreensão do fenômeno investigado.

 As informações provenientes das entrevistas foram analisadas qualitativamente por meio da análise temática de conteúdo, proposta por Bardin ^(^ 18 ^)^ . O processo analítico foi realizado em três fases: pré-análise, exploração do material e tratamento dos dados. Utilizou-se o *software* ATLAS.ti para as três etapas da análise de conteúdo, gerando 29 *codes* (unidades de registro) e 350 *quotations* (unidades de contexto). A partir destas unidades, foram realizadas a intersecção das interpretações dos dados e a categorização temática. 

### Aspectos éticos

O estudo foi aprovado pelo Comitê de Ética da Universidade Federal de Santa Catarina sob parecer n.º 5.256.168. Foram cumpridos todos os princípios apresentados pelas Resoluções n.º 466/2012 e n.º 510/2016 do Conselho Nacional de Saúde. Todas as etapas da pesquisa foram previamente explicadas aos participantes e esclarecida a participação facultativa em qualquer uma delas. Todos os participantes tiveram conhecimento e assinaram o Termo de Consentimento Livre e Esclarecido. Para a preservação da identidade, os participantes foram identificados com a letra R de residente ou P de preceptor e a respectiva sigla da categoria profissional à qual pertenciam. Quando houve mais de um participante da mesma profissão, foram enumerados com algarismos arábicos (1 ou 2).

## Resultados

 Participaram da pesquisa 146 preceptores e 58 residentes. Respeitou-se o Índice de Confiança (IC) de 95% e uma margem de erro de 5%. Com relação aos dados sociodemográficos, houve predominância de participantes do sexo feminino, tanto no grupo de preceptores quanto no grupo de residentes. A idade mínima dos participantes foi de 22 anos, e a máxima foi 62 anos. As oito profissões integrantes da RMS tiveram representação similar em termos de quantidade de participantes, excetuando-se a Enfermagem, que apresentou maior número quando comparado com todas as outras, uma vez que é a profissão com maior quantidade de vagas no programa. Na Tabela 1 é possível observar o detalhamento das características dos participantes. 

**Tabela 1:** - Características sociodemográficas dos residentes e preceptores de acordo com a frequência para variáveis categóricas e mediana para variáveis contínuas. Florianópolis, SC, Brasil, 2023

**Variável**	N * (%) ou mediana (mínimo-máximo)	
**Preceptores** (N * =146)	**Residentes** (N * =58)
**Idade**	39 [27,0 – 62,0]	25 [22,0 – 35,0]
**Sexo**		
Masculino	19 (13,0%)	12 (20,7%)
Feminino	127 (87,0%)	46 (79,3%)
**Profissão**		
Enfermagem	79 (54,1%)	16 (27,6%)
Fonoaudiologia	9 (6,2%)	03 (5,2%)
Psicologia	10 (6,8%)	11 (19,0%)
Farmácia	11 (7,5%)	7 (12,1%)
Serviço Social	13 (8,9%)	11 (19,0%)
Fisioterapia	8 (5,5%)	2 (3,4%)
Nutrição	12 (8,2%)	5 (8,6%)
Odontologia	4 (2,7%)	3 (5,2%)
**Área de concentração da RMS** ^†^		
Alta Complexidade	93 (63,7%)	37 (63,8%)
Urgência e Emergência	13 (8,9%)	11 (19,0%)
Saúde da Mulher e da Criança	40 (27,4 %)	10 (17,2%)
**Tempo de formado**		
Até 2 anos	NA ^‡^	26 (44,8%)
2 a 5 anos	NA ^‡^	30 (51,7%)
Mais de 5 anos	NA ^‡^	2 (3,4%)
**Ano da RMS** ^†^		
Primeiro ano (R1)	NA ^‡^	35 (60,3%)
Segundo ano (R2)	NA ^‡^	23 (39,7%)
**Experiência profissional**		
Até 2 anos	1 (0,7%)	NA ^‡^
3 e 5 anos	8 (5,5%)	NA ^‡^
6 e 10 anos	34 (23,3%)	NA ^‡^
Mais de 10 anos	103 (70,5%)	NA ^‡^
**Tempo de atuação como preceptor**		
Até 2 anos	27 (18,5%)	NA ^‡^
Entre 3 e 5 anos	53 (36,3%)	NA ^‡^
Entre 6 e 10 anos	43 (29,5%)	NA ^‡^
Mais de 10 anos	22 (15,1%)	NA ^‡^
Não respondeu	1 (0,6%)	NA ^‡^

* N = Amostra

^†^ RMS = Residência Multiprofissional em Saúde

^‡^ NA = Não se aplica

 Com relação à disponibilidade, todos os participantes se encaixaram nos percentis de moderada e alta disponibilidade, tanto para a EIP, avaliada pela RIPLS, como para a PC, avaliada pela EJARCI. Para preceptores, a proporção na RIPLS foi de 90,41% (132) com alta disponibilidade e 9,58% (14) com moderada disponibilidade; na EJARCI a proporção foi de 99,31% (145) com alta disponibilidade e 0,68% (1) com moderada disponibilidade. Para residentes, a proporção na RIPLS foi de 94,82% (55) com alta disponibilidade e 5,17% (3) com moderada disponibilidade; na EJARCI todos os 58 residentes atingiram alta disponibilidade. A Tabela 2 apresenta a pontuação global das duas escalas para residentes e preceptores. 

**Tabela 2:** - Pontuação dos Fatores 1, 2 e 3 da escala RIPLS * e pontuação global das escalas RIPLS * e EJARCI ^†^ . Florianópolis, SC, Brasil, 2023

	Dimensões RIPLS *			**Escore**	**Escore**
	**Fat 1** ^‡^	**Fat 2** ^§^	**Fat 3** ^||^	RIPLS *	**EJARCI** ^†^
**Preceptores (N** ^¶^ **=146)**					
Média	59,4	31,0	22,2	112,7	126,6
Desvio-padrão	5,5	2,9	2,2	8,2	8,4
Mediana	60,0	31,0	23,0	113,0	128,0
Pontuação mínima	43	22	16	89	100
Pontuação máxima	69	39	25	130	140
**Residentes (N** ^¶^ **=58)**					
Média	61,0	31,1	22,7	114,9	128,7
Desvio-padrão	4,0	2,9	2,2	6,9	7,6
Mediana	61,0	31,0	23,0	115,5	131,0
Pontuação mínima	51	23	17	95	108
Pontuação máxima	66	37	25	128	140

* RIPLS = *Readiness for Interprofessional Learning Scale*

^†^ EJARCI = Escala Jefferson de Atitudes Relacionadas à Colaboração Interprofissional

^‡^ Fat 1 **=** Fator 1

^§^ Fat 2 = Fator 2

^||^ Fat 3 = Fator 3

^¶^ N = Amostra

Na comparação entre preceptores de diferentes profissões, não houve diferenças sugestivas nas pontuações da RIPLS e da EJARCI, contudo, observou-se menor mediana na pontuação global das duas escalas nos preceptores enfermeiros (RIPLS = 112 e EJARCI = 122). O mesmo ocorreu quando foram realizados os testes comparando residentes de diferentes profissões, no entanto, as menores medianas observadas neste caso foram no Serviço Social para a RIPLS (109) e na Fonoaudiologia para a EJARCI (123).

 Especificamente para o tempo de formado, houve diferença sugestiva (P < 0,014) para o Fator 1 da RIPLS - Trabalho em Equipe e Colaboração. No teste *post hoc* , a diferença foi encontrada entre os residentes com tempo de formado de até 2 anos e entre aqueles com mais de 5 anos. 

 Na comparação das pontuações globais entre preceptores e residentes de uma mesma profissão, os resultados indicam diferenças em duas profissões, conforme pode ser observado na Tabela 3 . 

**Tabela 3:** - Comparação da pontuação global nas escalas RIPLS * e EJARCI ^†^ de acordo com preceptores e residentes de uma mesma profissão. Florianópolis, SC, Brasil, 2023

**Profissão**	Dimensões da RIPLS *			**Escore**	
	**Fat 1** ^‡^	**Fat 2** ^§^	**Fat 3** ^||^	RIPLS *	**EJARCI** ^†^
**ENF** ^¶^					
Preceptores	61,0 [45,0 – 67,0]	32,0 [24,0 – 39,0]	22,0 [16,0 – 25,0]	113,0 [96,0 – 130,0]	129,0 [100,0 – 140,0]
Residentes	60,0 [54,0 – 66,0]	32,0 [23,0 – 36,0]	22,5 [17,0 – 25,0]	118,0 [95,0 – 125,0]	131,5 [112,0 – 140,0]
*P* valor **	0,575	0,320	0,884	0,417	0,322
**FAM** ^††^					
Preceptores	60,0 [54,0 – 69,0]	31,0 [27,0 – 34,0]	22,0 [18,0 – 25,0]	111,0 [105,0 – 122,0]	126,0 [111,0 – 137,0]
Residentes	60,0 [51,0 – 66,0]	30,0 [26,0 – 34,0]	23,0 [18,0 – 25,0]	112,0 [101,0 – 125,0]	122,0 [108,0 – 134,0]
*P* valor **	0,930	0,596	0,479	0,930	0,536
**FSI** ^‡‡^					
Preceptores	61,0 [56,0 – 66,0]	31,0 [28,0 – 37,0]	23,5 [20,0 – 25,0]	117,5 [107,0 – 123,0]	130,0 [110,0 – 140,0]
Residentes	64,5 [63,0 – 66,0]	32,0 [30,0 – 34,0]	22,5 [20,0 – 25,0]	119,0 [113,0 – 125,0]	129,0 [124,0 – 134,0]
*P* valor **	0,400	0,711	1,000	0,533	1,000
**FON** ^§§^					
Preceptores	58,0 [51,0 – 66,0]	31,0 [27,0 – 33,0]	23,0 [20,0 – 25,0]	112,0 [106,0 – 122,0]	124,0 [120,0 – 137,0]
Residentes	64,0 [64,0 – 65,0]	35,0 [29,0 – 35,0]	24,0 [23,0 – 24,0]	122,0 [117,0 – 124,0]	134,0 [133,0 – 139,0]
*P* valor **	0,064	0,373	0,373	**0,018**	**0,036**
**NUT** ^||||^					
Preceptores	56,0 [43,0 – 66,0]	29,5 [27,0 – 25,0]	22,5 [19,0 – 25,0]	110,0 [89,0 – 119,0]	123,0 [111,0 – 135,0]
Residentes	64,0 [59,0 – 66,0]	31,0 [29,0 – 35,0]	25,0 [21,0 – 25,0]	120,0 [116,0 – 123,0]	133,0 [122,0 -140,0]
*P* valor **	**0,004**	0,442	0,082	**0,001**	**0,037**
**ODO** ^¶¶^					
Preceptores	60,5 [58,0 – 66,0]	31,0 [29,0 – 33,0]	21,5 [20,0 – 23,0]	112,5 [109,0 – 121,0]	124,0 [118,0 – 131,0]
Residentes	60,0 [55,0 – 61,0]	32,0 [30,0 – 34,0]	25,0 [20,0 – 25,0]	114,0 [110,0 – 118,0]	139,0 [133,0 – 140,0]
*P* valor **	0,629	0,629	0,400	0,857	0,057
PSI ***					
Preceptores	63,0 [49,0 – 66,0]	32,5 [29,0 – 35,0]	24,0 [21,0 – 25,0]	119,0 [103,0 – 123,0]	127,0 [116,0 – 138,0]
Residentes	61,0 [57,0 – 66,0]	31,0 [27,0 – 37,0]	23,0 [20,0 – 25,0]	113,0 [109,0 – 128,0]	126,0 [117,0 – 135,0]
*P* valor **	0,173	0,314	0,468	0,223	0,756
**SSO** ^†††^					
Preceptores	60,00 [49,0 – 66,0]	28,0 [22,0 – 35,0]	23,0 [20,0 – 25,0]	109,0 [98,0 – 125,0]	125,0 [109,0 – 140,0]
Residentes	60,0 [55,0 – 66,0]	28,0 [26,0 – 33,0]	23,0 [19,0 – 25,0]	115,0 [100,0 – 124,0]	130,0 [111,0 – 139,0]
*P* valor **	0,733	0,733	0,820	0,361	0,569

* RIPLS = *Readiness for Interprofessional Learning Scale*

^†^ EJARCI = Escala Jefferson de Atitudes Relacionadas à Colaboração Interprofissional

^‡^ Fat 1 **=** Fator 1

^§^ Fat 2 = Fator 2

^||^ Fat 3 = Fator 3

^¶^ ENF = Enfermagem

** Teste U de Mann Whitney

^††^ FAM = Farmácia

^‡‡^ FIS = Fisioterapia

^§§^ FON = Fonoaudiologia

^||||^ NUT = Nutrição

^¶¶^ ODO = Odontologia

*** PSI = Psicologia

^†††^ SSO = Serviço Social

A hipótese nula adotada foi de que a distribuição da pontuação seria a mesma entre residentes e preceptores. Rejeitou-se essa hipótese para Fonoaudiologia na pontuação global da RIPLS (U= 1,500; p < 0,018) e na pontuação global da EJARCI (U= 2,500; p < 0,036). Rejeitou-se também para Nutrição na pontuação do Fator 1 da RIPLS (U= 4,500; p < 0,004), pontuação global da RIPLS (U= 2,500; p < 0,001) e pontuação global da EJARCI (U= 10,500; p < 0,037).

A análise temática indicou que o processo de formação na graduação e a resistência de alguns profissionais são duas das principais oportunidades de melhorar a disponibilidade. Já a disposição para o diálogo horizontal e a colaboração da equipe multiprofissional, tanto entre preceptores como residentes, foram evidenciadas como as principais potencialidades.

A formação dos profissionais, em sua maioria na perspectiva uniprofissional, desempenha papel limitador para as práticas interprofissionais, em função da falta de reconhecimento das funções dos colegas de outra categoria profissional, como pode ser observado nos depoimentos.

*Ainda estamos nos encaminhando para isso* [práticas interprofissionais] *, não é algo específico daqui, mas decorre da nossa formação também, que é só voltada para a nossa categoria profissional, não temos conhecimento da parte técnica dos outros profissionais para que eles tomem as condutas que vemos eles tomando.* (P.FON) 

*Os próprios professores têm dificuldade de colocar nossa profissão nas aulas em um contexto interprofissional. Eu até entendo, porque apesar de sermos todas profissões da saúde, somos formados separados e existem segmentos diferentes dentro de cada profissão, então entendo a dificuldade.* (R.ODO1) 

*Como dificuldade, principalmente nas aulas, eu tenho por não ter formação clínica, nós não vemos nada sobre o corpo* [anatomia, fisiologia etc.] *, a nossa formação é isolada de todos os outros profissionais da saúde. Então às vezes eu nem entendo do que estão falando, a maioria das coisas eu preciso ficar perguntando do que se trata.* (R.SSO1) 

*Temos dificuldades de ter relações com outros profissionais, por estar muito restrito à nossa área. Na Odontologia, infelizmente, temos uma formação que não envolve outros profissionais, então temos como consequência essa dificuldade de saber como trabalhar com todo mundo para suprir as necessidades dos pacientes.* (R.ODO2) 

*Eu vejo que acabamos tentando explicar a todo momento ao que a gente veio. Eu vejo mais que a Psicologia e o Serviço Social precisam muito provar o porquê estão ali, as pessoas têm dificuldade para demandar o Serviço Social ou a Psicologia, muito em função da formação na graduação também, que é mais isolada das outras profissões.* (R.PSI1) 

Com relação ao perfil profissional, as influências ocorrem devido à resistência de alguns profissionais em desenvolver a colaboração tanto para o ensino como para o trabalho. Há receio de compartilhar informações e tendência em agir de forma autônoma, principalmente quando envolve estudantes ou residentes.

*Tem muita resistência do pessoal, é muito... não sei se é a palavra certa, mas egoísmo. Tem algumas profissões que percebemos que eles não querem compartilhar o conhecimento porque têm medo de que os outros saibam mais do que eles, isso desanima a gente, como vamos aprender juntos deste jeito?* (R.FAM1) 

*Tem colega enfermeiro que falava “eu não gosto de aluno, não quero ninguém da residência comigo”. Esses profissionais faziam as coisas sem os estudantes, para fazer mais rápido, o que acabava deixando-os sem vontade de aprender.* (P.ENF1) 

*Vemos quem está de plantão, procuramos uma pessoa que já conhecemos, que sabemos que tem uma postura mais amigável, mais sensibilidade, mais colaborativo. Às vezes eu prefiro não discutir o caso dependendo de quem é o outro profissional, porque sei que não vai evoluir, não vai me escutar.* (P.SSO) 

Em contrapartida, o acesso facilitado e o diálogo horizontal influenciam no desejo de querer aprender a trabalhar de modo colaborativo na disponibilidade. O diálogo efetivo e a paridade no peso das contribuições na equipe multiprofissional sustentam a vontade de atuar colaborativamente.

*Por isso a importância de ter um bom acesso à equipe. Na UTI NEO eu consigo dialogar com o médico o tempo todo, com a enfermagem. A equipe tem uma sistemática de reunião para discussão de casos e das condutas e não somente para discussão das condutas, mas discussão das mudanças.* (P.SSO) 

*Aqui eu senti que conseguimos nos comunicar com a equipe e traçar objetivos junto com a equipe. A equipe multiprofissional da reabilitação, fisio, fono, nutri, psicólogo e a enfermagem, o que nos motiva a querer compartilhar ideias.* (P.FIS1)**

*Então, os médicos, enfermeiros e outros profissionais chegam até mim para perguntar e trocar ideias. É bem aberto e tranquilo, é um diálogo bom* . (R.FIS) 

*Na UTI NEO, é muito discutido cada caso com todas as profissões, tem todo um desenvolvimento conjunto, nenhuma decisão é tomada sem ser conversada com a fono, enfermagem, serviço social. Tudo é muito conversado. Enxergamos o quanto o outro é importante.* (R.ENF2) 

*Tem pessoas que se dispõem mais, são mais acessíveis, mais fáceis de lidar, e com essas pessoas o trabalho flui.* (P.ENF1) 

A formação de graduação exerce influência no desenvolvimento de práticas interprofissionais, nas perspectivas dos residentes e dos preceptores do Serviço Social e da Psicologia, que relataram dificuldades para atuar na equipe multiprofissional, devido às limitações de compreensão dos aspectos biológicos do cuidado e ao não reconhecimento da importância dos aspectos psicossociais por parte das outras profissões, como por exemplo, a Odontologia, cuja formação é direcionada aos aspectos anatômicos e fisiopatológicos da boca, e não há tanta predisposição ao diálogo com estudantes de outras profissões da área da saúde.

 Para a integração e a compreensão dos dados, utilizou-se a técnica de *joint-display* , apresentado na Figura 1 . 

**Figura 1:** - *Joint-display* para a integração dos dados quantitativos e qualitativos. Florianópolis, SC, Brasil, 2023

*M _e_ = Média; ^†^ EIP = Educação Interprofissional; ^‡^ PC = Prática Colaborativa; ^§^ Teste U de Mann-Whitney; ^||^ FON = Fonoaudiologia; ^¶^ NUT = Nutrição; **Maior frequência de respostas; ^††^ Teste Kruskal-Wallis

## Discussão

 As práticas interprofissionais desempenham um papel importante ao fomentar o compartilhamento de experiências entre equipes multiprofissionais e entre residentes de diferentes profissões, promovendo a centralidade do cuidado no paciente e a convergência das áreas do conhecimento ^(^ 1 ^-^ 3 ^)^ . No contexto deste estudo, os achados indicam alta disponibilidade dos participantes para o desenvolvimento das práticas interprofissionais nos âmbitos educacionais e de cuidado. 

 Destaca-se que essa alta disponibilidade pode estar relacionada ao contexto em que os participantes estão inseridos, especialmente considerando o ambiente de hospitais universitários, propícios à implementação de inovações na prestação de cuidados, devido às suas conexões com universidades, o que fomenta o ensino e a pesquisa ^(^ 19 ^)^ . Adicionalmente, a RMS oferece uma abordagem diferenciada de pós-graduação, permitindo aos residentes e preceptores a oportunidade de aprendizado e prática simultâneos, e incentivando a colaboração entre profissionais de diversas áreas ^(^ 8 ^-^ 9 ^,^ 19 ^)^ . Assim, a sensibilização para a importância da colaboração no ensino e no cuidado pode ser intensificada, promovendo disposição para as práticas interprofissionais. 

 Apesar disso, os participantes indicam que a disposição por si só não é suficiente para implementar práticas interprofissionais consistentes na RMS hospitalar. Isso pode estar relacionado às rotinas hospitalares, que ainda permanecem rígidas, com papéis profissionais delimitados por fronteiras historicamente definidas, muitas vezes marcadas pela ausência de autonomia ou por responsabilidades relacionadas à execução técnica de procedimentos. O predomínio do modelo biomédico ainda coloca o diagnóstico e a cura como foco, resultando na supervalorização das práticas médicas e na menor autonomia e reconhecimento dos demais profissionais, o que impacta negativamente a valorização do trabalho colaborativo ^(^ 19 ^)^ . 

 O modelo biomédico mantém sua histórica influência na área da saúde, não apenas nos cuidados clínicos, mas também na formação. Esse estudo destaca que a formação na graduação, exceto por algumas iniciativas isoladas, frequentemente mantém uma abordagem disciplinar e uniprofissional do conhecimento, o que pode limitar as oportunidades para os estudantes aprenderem uns com os outros e entre si ^(^ 6 ^)^ . A falta de sensibilização para a colaboração na graduação dificulta a formação de profissionais colaborativos. Além disso, essa lacuna pode perpetuar um ciclo de resistência ou adoção parcial de práticas interprofissionais, especialmente à medida que estes estudantes tenham dificuldades em integrar conhecimentos e ações interprofissionais nos futuros locais de atuação ^(^ 20 ^)^ . 

 Contudo, atribuir à formação na graduação parte das dificuldades de trabalhar de maneira interprofissional pode mascarar as dificuldades de entender os limites estabelecidos dentro de cada área profissional, o que parece ser mais definido na Medicina, em comparação às demais profissões ^(^ 21 ^)^ . A ideia de precisar aprender a profissão do outro para, então, contribuir interprofissionalmente, conflita com a necessidade de múltiplos profissionais em suas próprias formações trabalharem juntos, cada um em seu escopo, para então colaborarem entre si ^(^ 5 ^)^ . Outro ponto crítico para o desenvolvimento da EIP e da PC, como indicado pelos participantes deste estudo, são determinadas características profissionais, especialmente com relação à comunicação, ferramenta essencial na área da saúde ^(^ 2 ^,^ 22 ^)^ . Uma comunicação ineficaz e a falta de abertura para o diálogo interprofissional comprometem o ensino e o cuidado, perpetuando a fragmentação das atividades assistenciais e do conhecimento. 

 Considerando uma abordagem baseada em competências para a prática interprofissional, a comunicação emerge como um atributo fundamental ^(^ 23 ^-^ 24 ^)^ . A comunicação efetiva, empática e não-violenta não apenas facilita a colaboração interprofissional, mas também cria ambientes de aprendizado saudáveis e práticas de cuidados seguros, minimizando erros e promovendo o cuidado ^(^ 24 ^)^ . Nesse contexto, habilidades de comunicação demandam treinamento e aprimoramento, possibilitando mudanças positivas nas atitudes e comportamentos dos profissionais e estimulando o diálogo constante e acolhedor no âmbito da RMS. 

Este estudo contribui significativamente para o avanço do conhecimento na área da saúde, abordando uma temática globalmente relevante. Para efetivar práticas interprofissionais, é essencial, dentre outras ações, mensurar a disponibilidade e compreender os fatores que influenciam a adesão de profissionais e estudantes a essa abordagem. A RMS surge como uma ferramenta promissora para a incorporação dessas práticas no contexto hospitalar brasileiro, justificando plenamente sua investigação.

Dentre as limitações deste estudo, aponta-se a ausência de professores e tutores da RMS como participantes, bem como de pacientes e familiares, cuja inclusão poderia enriquecer a compreensão do fenômeno, especialmente no âmbito qualitativo. Além disso, a inclusão de profissionais e residentes da Medicina proporcionaria uma visão abrangente das dinâmicas interprofissionais e seu impacto na disposição para a colaboração, pois avaliar os profissionais da residência multiprofissional sem considerar a figura do médico na abordagem ao paciente indica um viés de seleção, afinal, as ações da equipe multiprofissional incluem abordagens de todas as profissões da área da saúde. Ademais, residentes e preceptores com atitudes mais positivas em relação à EIP podem ter sido mais propensos a participar do estudo, o que pode ter influenciado os resultados.

## Conclusão

O estudo apresentou a disponibilidade para o desenvolvimento de práticas interprofissionais de residentes e preceptores de uma Residência Multiprofissional em Saúde hospitalar. Os resultados quantitativos indicaram alta disponibilidade para a Educação Interprofissional e a Prática Colaborativa entre os participantes e, a partir da integração dos dados coletados de maneira concomitante, os resultados qualitativos possibilitaram a compreensão dos fatores envolvidos nesta disponibilidade para a EIP e a PC.

Apesar disso, assinala-se como dificuldade para o desenvolvimento e manutenção das práticas interprofissionais a formação na graduação e as características dos profissionais envolvidos, tais como as dificuldades de comunicação entre as profissões e de abertura ao diálogo. Estas dificuldades podem e devem ser observadas sob a perspectiva de oportunidades, buscando-se aperfeiçoamento das habilidades de comunicação dos residentes, preceptores e profissionais em geral, com vistas à melhoria dos processos de ensino e de trabalho no âmbito das Residências Multiprofissionais em Saúde.
